# The Genus *Leccinum*: Global Advances in Taxonomy, Ecology, Nutritional Value, and Environmental Significance

**DOI:** 10.3390/jof12010070

**Published:** 2026-01-16

**Authors:** Ruben Budau, Simona Ioana Vicas, Mariana Florica Bei, Danut Aurel Dejeu, Lucian Dinca, Danut Chira

**Affiliations:** 1 Department of Silviculture and Forestry Engineering, Environmental Protection Faculty, University of Oradea, General Magheru Boulevard 26, 410048 Oradea, Romania; rbudau@uoradea.ro; 2 Department of Food Engineering, Environmental Protection Faculty, University of Oradea, General Magheru Boulevard 26, 410048 Oradea, Romania; svicas@uoradea.ro; 3 Departament Discipline Chirurgicale, Faculty of Medicine and Pharmacy, University of Oradea, Piata 1 Decembrie 10, 410073 Oradea, Romania; ddejeu@uoradea.ro; 4 National Institute for Research and Development in Forestry “Marin Dracea”, Eroilor 128, 077190 Voluntari, Romania; danutchira61@gmail.com

**Keywords:** Boletaceae, ectomycorrhizal fungi, forest ecology, mycorrhizal associations, host specificity, species diversity, biogeography

## Abstract

*Leccinum* is an ecologically significant and taxonomically complex genus of ectomycorrhizal fungi widely distributed across boreal, temperate, Mediterranean, and selected tropical regions. Despite its ecological, nutritional, and applied importance, no comprehensive review has previously synthesized global knowledge on this genus. This work provides the first integrative assessment of *Leccinum* research, combining a bibliometric analysis of 293 peer-reviewed publications with an in-depth qualitative synthesis of ecological, biochemical, and environmental findings. Bibliometric results show increasing scientific attention since the mid-20th century, with major contributions from Europe, Asia, and North America, and dominant research themes spanning taxonomy, ecology, chemistry, and environmental sciences. The literature review highlights substantial advances in phylogenetic understanding, species diversity, and host specificity. *Leccinum* forms ectomycorrhizal associations with over 60 woody host genera, underscoring its functional importance in forest ecosystems. Nutritionally, *Leccinum* species are rich in proteins, carbohydrates, minerals, bioactive polysaccharides, phenolic compounds, and umami-related peptides, with demonstrated antioxidant, immunomodulatory, and antitumor activities. At the same time, the genus exhibits notable bioaccumulation capacity for heavy metals (particularly Hg, Cd, and Pb) and radionuclides, making it both a valuable food source and a sensitive environmental bioindicator. Applications in biotechnology, environmental remediation, forest restoration, and functional food development are emerging but remain insufficiently explored. Identified research gaps include the need for global-scale phylogenomic frameworks, expanded geographic sampling, standardized biochemical analyses, and deeper investigation into physiological mechanisms and applied uses. This review provides the first holistic synthesis of *Leccinum*, offering an integrated perspective on its taxonomy, ecology, nutritional composition, environmental significance, and practical applications. The findings serve as a foundation for future mycological, ecological, and biotechnological research on this diverse and understudied fungal genus.

## 1. Introduction

The genus *Leccinum* Gray (1821) comprises a morphologically distinctive and ecologically important group of ectomycorrhizal fungi widely distributed across temperate, boreal, subarctic, and Mediterranean regions of the Northern Hemisphere, with additional records from the Neotropics [1,2,3,4]. Current estimates recognize approximately 150 species within *Leccinum*, although species richness and circumscription have varied considerably over time due to taxonomic instability and evolving generic concepts [5,6,7].

Historically, *Leccinum* served as a broad taxonomic repository for numerous “leccinoid” taxa within the Boletaceae, many of which have since been segregated into distinct genera such as *Leccinellum*, *Hemileccinum*, *Rossbeevera*, and *Chamonixia* [8,9,10,11]. Classical morphological studies emphasized characters such as scabrous stipes, hymenophore color changes, and host association, but also documented substantial overlap among taxa, complicating species delimitation [2,5,12]. Recent molecular phylogenetic analyses, often combined with detailed morphological reassessment, have clarified generic boundaries and revealed cryptic diversity within *Leccinum*, particularly in North America and East Asia [3,13].

Ecologically, *Leccinum* species are obligate ectomycorrhizal fungi exhibiting relatively strong host specificity compared with many other boletoid genera. Most species associate with a limited range of woody hosts, particularly *Betula*, *Populus*, *Salix*, *Quercus*, and *Fagus*, making *Leccinum* a valuable model for studying host–fungus specificity, co-evolution, and forest biogeography [5,7,13]. Through these symbiotic relationships, *Leccinum* species contribute to nutrient acquisition, carbon allocation belowground, and overall forest ecosystem functioning. In addition to their symbiotic role, *Leccinum* fruiting bodies support a diverse assemblage of mycetophilous organisms, including insects, gastropods, mammals, bacteria, and fungi, thereby contributing to forest food webs and biodiversity [14]. From a socio-economic perspective, *Leccinum* species rank among the most widely collected and consumed wild mushrooms in boreal and temperate regions. Species such as *Leccinum scabrum* are particularly important in traditional diets and local markets, where they represent a seasonal source of nutrition and income [14,15].

Recent studies have highlighted the nutritional and medicinal potential of *Leccinum* species. Chemical analyses demonstrate that *Leccinum* fruiting bodies contain high-quality proteins, dietary fiber, essential minerals, and vitamins [16]. Species-specific investigations have identified polysaccharides and other bioactive compounds with antioxidant, antimicrobial, and immunomodulatory properties, suggesting potential applications in functional foods and nutraceuticals [17,18]. Importantly, these properties vary substantially among *Leccinum* species, reinforcing the need for accurate taxonomy and comparative studies within the genus.

Another notable characteristic of *Leccinum* is its capacity to bioaccumulate trace elements and metals from soil substrates. This trait has been explored in ecological and applied contexts, including the use of *Leccinum* species as indicators of soil chemistry and environmental contamination, as well as their potential relevance in geobotanical studies [15,18].

Climate change modifies the precipitation patterns, increases the temperature and amplifies the frequency and severity of extreme events [19,20]. Altered temperature and precipitation regimes can cause drought-induced tree mortality, increased soil erosion, and reduced forest capability to provide essential ecosystem services functions [21,22,23,24,25,26,27,28]. These changes in temperature, precipitation, water availability and forestry composition represent a major stressor for host trees and will directly alter the distribution and abundance of the associated *Leccinum* species.

Over the past several decades, research on *Leccinum* has expanded considerably, encompassing taxonomy, phylogenetics, ecology, chemistry, physiology, biogeography, and conservation [3,4,13,29,30].

Despite the extensive body of literature devoted to individual mushroom species and economically important fungal groups, no comprehensive review has yet synthesized the scientific knowledge related to the genus *Leccinum*. This absence is notable given the genus’s ecological significance, taxonomic complexity, wide geographic distribution, and long-standing relevance to human use. Species of *Leccinum* are prominent ectomycorrhizal fungi in forest ecosystems, play key roles in nutrient cycling and plant–fungus symbioses, and are widely collected for food across Europe, Asia, and North America.

The rapid expansion of research on fungal taxonomy, molecular phylogenetics, environmental monitoring, and bioactive compounds has generated a fragmented but substantial literature on *Leccinum*. However, these studies remain dispersed across disciplines, limiting their integration into a unified framework that connects ecological function, chemical composition, environmental sensitivity, and practical applications. A consolidated synthesis is therefore needed to evaluate existing knowledge, identify trends and gaps, and support future research and applied management.

The primary objective of this review is to provide the first holistic and multidisciplinary synthesis of global scientific research on the genus *Leccinum*. Specifically, this article aims to:

(1) Analyze global research output on *Leccinum* through a bibliometric assessment of publication trends, geographic distribution, major contributors, and thematic evolution;
(2) Synthesize current knowledge on *Leccinum* taxonomy, phylogeny, species diversity, biogeography, host specificity, and ecological roles in forest ecosystems;
(3) Evaluate the nutritional composition and bioactive compounds of *Leccinum* species, with emphasis on their relevance for human consumption, functional foods, and potential medicinal applications;
(4) Assess the capacity of *Leccinum* species to accumulate mineral elements, heavy metals, and radionuclides, and discuss their implications for environmental monitoring and food safety;
(5) Examine documented and potential uses of *Leccinum* in forest symbiosis, ecological restoration, biotechnology, and therapeutic research; and
(6) Identify critical knowledge gaps and future research directions necessary to refine taxonomic frameworks, improve ecological understanding, and expand the applied and environmental potential of the genus.

By integrating ecological, biochemical, environmental, and applied perspectives, this review seeks to provide a coherent reference for mycologists, ecologists, food scientists, and forest managers, and to support evidence-based utilization and conservation of *Leccinum* species worldwide.

## 2. Materials and Methods

The methodological workflow of this review comprised two complementary components. The first component involved a bibliometric assessment aimed at identifying global research trends, authorship patterns, and thematic concentrations in studies concerning the genus *Leccinum*. The second component consisted of a traditional literature review designed to synthesize qualitative insights from the selected publications, thereby contextualizing quantitative findings within broader ecological and mycological knowledge.

### 2.1. Bibliometric Analysis

Data Sources and Search Strategy

The bibliometric analysis drew upon two major international bibliographic databases: Scopus and the Science Citation Index Expanded (SCI-Expanded) within the Web of Science (WoS) platform. Searches were performed using the core keyword “*Leccinum*,” supplemented with additional subject-specific terms to capture ecological, physiological, and forest-related aspects. Boolean operators (AND, OR) and wildcard characters (*) were applied to construct a comprehensive search strategy encompassing both broad and specific aspects of *Leccinum* research.

All retrieved records were screened and refined following the PRISMA (Preferred Reporting Items for Systematic Reviews and Meta-Analyses) framework to ensure methodological transparency and reproducibility [31].

Search Strings

To ensure reproducibility, the exact search strings applied in each database are detailed below:

Scopus (Advanced Search; field: TITLE-ABS-KEY):

TITLE-ABS-KEY(“Leccinum” AND (“forest dynamics” OR “mycorrhizal associations” OR “ecology” OR “soil interactions” OR “nutrient cycling”))

Web of Science—SCI-Expanded (Topic Search; TS):

TS = (“Leccinum” AND (“forest dynamics” OR “mycorrhizal interactions” OR “ecology” OR “nutrient cycling” OR “soil chemistry”))

Wildcard characters (*) were applied to capture plural forms and morphological variations. Minor syntax adjustments were made between databases to maintain logical consistency and accommodate platform-specific search rules.

Search Parameters

Time range: All available years up to the date of data extraction; no temporal restrictions were imposed.

Document types: Only peer-reviewed original research articles and review papers were included.

Excluded records: Conference proceedings, editorials, letters, notes, theses, and book chapters were removed.

De-duplication and Data Validation

Duplicate records were removed through a two-step procedure:

1. Automated removal using DOI and title matching in Microsoft Excel.
2. Manual verification of residual duplicates by comparing titles, authors, publication years, and journal information.

This process eliminated 189 duplicate entries. Additional quality control included verification of bibliographic metadata, correction of typographical or OCR errors, and harmonization of author and institutional names. All modifications were documented in an internal audit log.

Eligibility Criteria

A two-stage screening process was employed:

Stage 1—Title and abstract screening: Conducted independently by two reviewers.

Stage 2—Full-text evaluation: Applied to all potentially relevant articles.

Inclusion criteria:

- Peer-reviewed publications.
- Direct focus on *Leccinum* in relation to forest ecosystems, mycorrhizal interactions, or ecological processes.
- Sufficient methodological description and complete bibliographic metadata.

Exclusion criteria:

- Non-peer-reviewed documents (editorials, correspondence, theses).
- Studies unrelated to forest ecosystems or without relevant ecological data.
- Records with inaccessible full texts or missing abstracts.

Full-text exclusions were coded as follows:

A: Outside thematic scope

B: Non-peer-reviewed

C: Insufficient ecological or methodological data

D: Inaccessible full text

E: Inadequate methodological description

Screening Process

Screening was conducted independently by two reviewers, with any record deemed relevant by at least one reviewer progressing to full-text review. Discrepancies were resolved through consultation with a senior reviewer.

**Figure 1:**
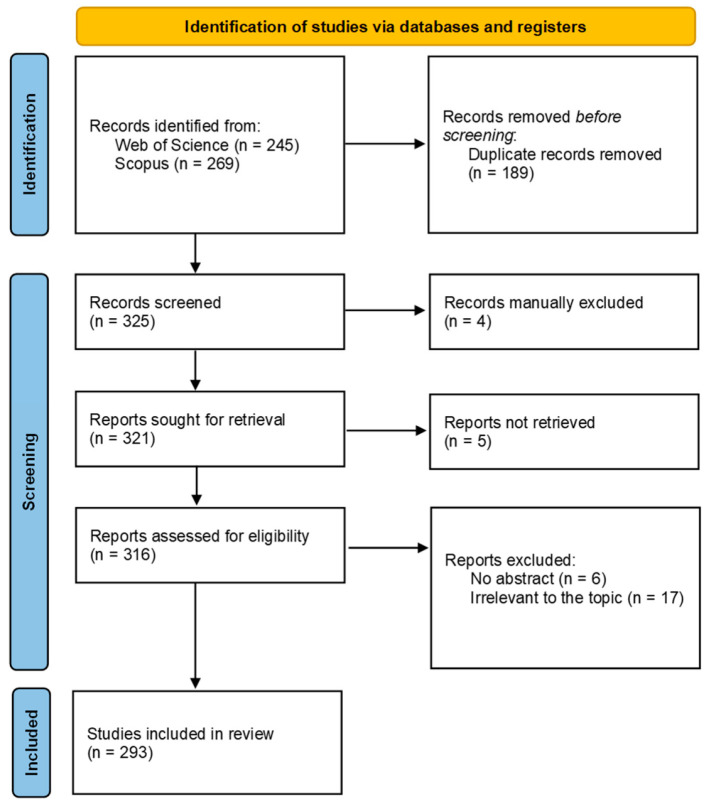
Selection process of the eligible reports based on the PRISMA 2020 flow diagram.

The initial search yielded 514 records (269 from Scopus, 245 from WoS). After removing 70 duplicates, 444 unique records remained. Following two-stage screening based on the above criteria, an additional 151 records were excluded due to irrelevance, non-peer-review status, or lack of accessible abstracts, resulting in a final dataset of 293 publications for bibliometric and content analyses (Figure 1).

Bibliometric Analysis Tools

Bibliometric analyses were conducted using Scopus [32], Web of Science Core Collection (v.5.35) [33], Microsoft Excel 2024 [34], and Geochart [35]. Network visualizations for co-authorship, co-citation, and keyword co-occurrence analyses were constructed using VOSviewer (v.1.6.20) [36].

All fungal names and their current taxonomic status were verified using Index Fungorum. The nomenclature of vascular plants was standardized primarily according to Flora Europaea and the Euro+Med PlantBase. However, to maintain consistency with the original literature and avoid misinterpretation, the plant names used by the original authors were retained where appropriate.

### 2.2. Traditional Literature Review

The second phase involved a narrative synthesis based on the refined dataset. A subset of 256 publications underwent detailed qualitative assessment to identify dominant research themes, conceptual linkages, and knowledge gaps. The literature was organized into seven overarching thematic domains: (1) *Leccinum* species and their characteristics; (2) Tree and plant species with *Leccinum*; (3) Nutritional content of *Leccinum*; (4) Mineral constituents of *Leccinum*; (5) Heavy metals content in *Leccinum*; (6) Reactivity content in *Leccinum*; (7) Utilization of *Leccinum*.

**Figure 2:**
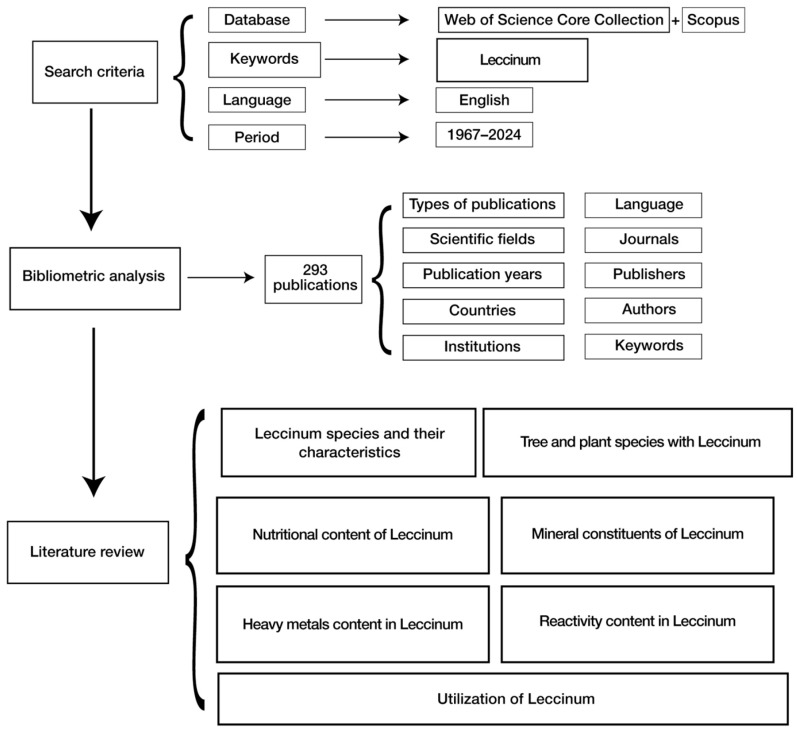
Schematic presentation of the workflow used in our research.

A schematic overview of the complete methodological framework is provided in Figure 2, summarizing the workflow from initial data acquisition through quantitative bibliometric analysis and final qualitative synthesis.

The qualitative content analysis enabled a deeper understanding of research production and thematic trends, complementing the bibliometric assessment and providing a comprehensive view of global *Leccinum* research.

This study considers only species currently accepted within the genus *Leccinum*, based on recent taxonomic revisions; taxa formerly included in *Leccinum* but now transferred to other genera (e.g., *Leccinellum, Rugiboletus*, etc.) are not included.

Although no temporal restriction was imposed during the literature search, the earliest publication retrieved that met the inclusion criteria dated from 1967; therefore, the analyzed literature spans the period from 1967 to the present.

## 3. Results

### 3.1. A Bibliometric Review

**Figure 3:**
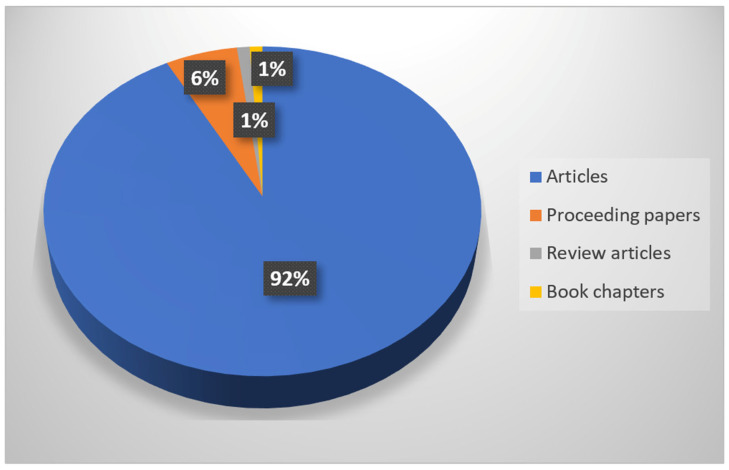
Types of publications on *Leccinum*.

The inventory of published documents on this topic from 1967–2024 resulted in a total of 293 publications. Of these, most are research articles (270, representing 92% of the total), followed by 17 proceedings papers (6%), 3 reviews (1%), and 3 book chapters (1%) (Figure 3).

**Figure 4:**
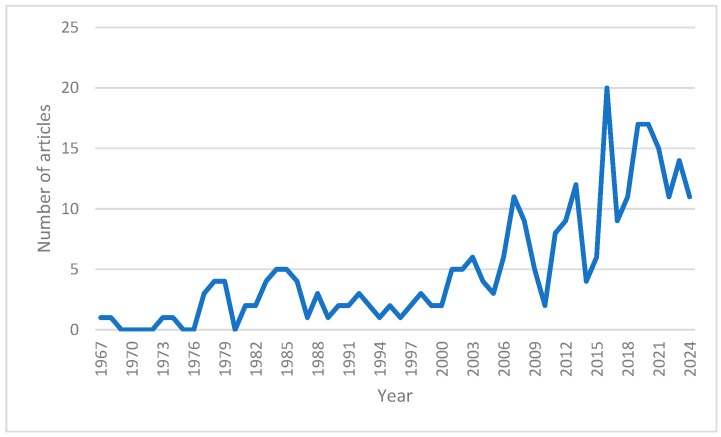
Representation of the number of publications by year on *Leccinum*.

Starting from 1967, the number of published articles has fluctuated considerably up to the present year (2024). The highest number of publications (20 articles) was recorded in 2016, while over the past seven years (2018–2024), the number has remained relatively constant, averaging around 15 articles per year (Figure 4).

**Figure 5:**
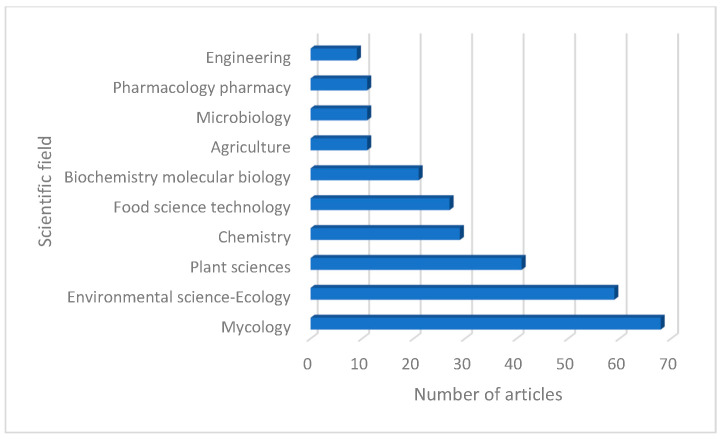
Distribution of the primary research areas in publications on *Leccinum*.

Among the 35 research areas in which these articles can be classified, the most representative are: Mycology (with 68 articles), Environmental sciences—Ecology (with 59 articles), Plant sciences (with 41 articles), and Chemistry (with 29 articles) (Figure 5).

**Figure 6:**
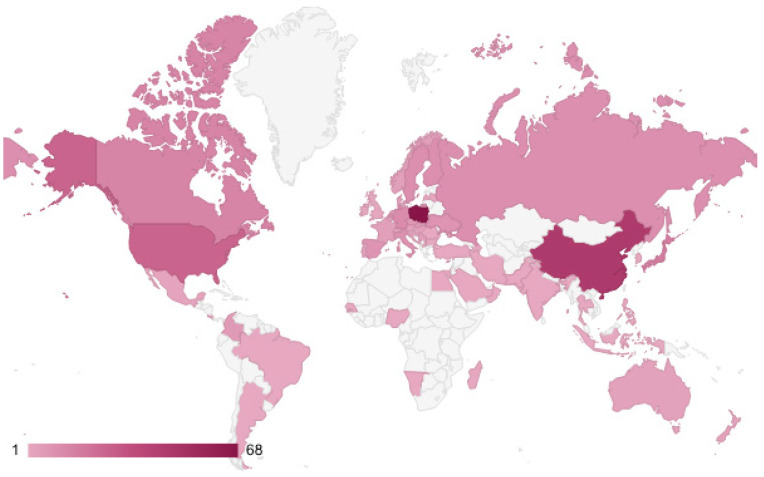
Countries with authors of articles on *Leccinum*.

Authors of articles on this topic come from 62 countries across 5 continents (Figure 6). The countries with the highest number of publications are Poland (63 articles), China (45 articles), the USA (26 articles), and Japan (17 articles).

**Figure 7:**
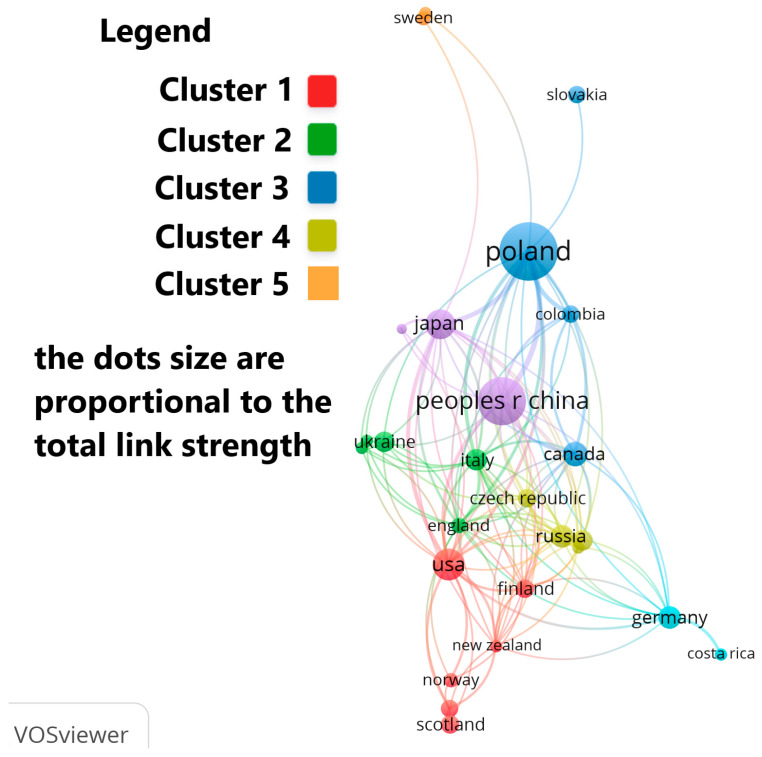
Clusters of countries with authors of articles on *Leccinum*.

The authors’ countries of origin, who have published articles on this topic, can be organized into four main clusters: Cluster 1 includes: Finland, Netherlands, New Zealand, Norway, Scotland and USA; Cluster 2 consists of: Austria, England, Hungary, Italy and Ukraine; Cluster 3 consists of: Canada, Columbia, Poland and Slovakia; and Cluster 4 includes: Czech Republic, Portugal, Russia and Spain (Figure 7).

**Figure 8:**
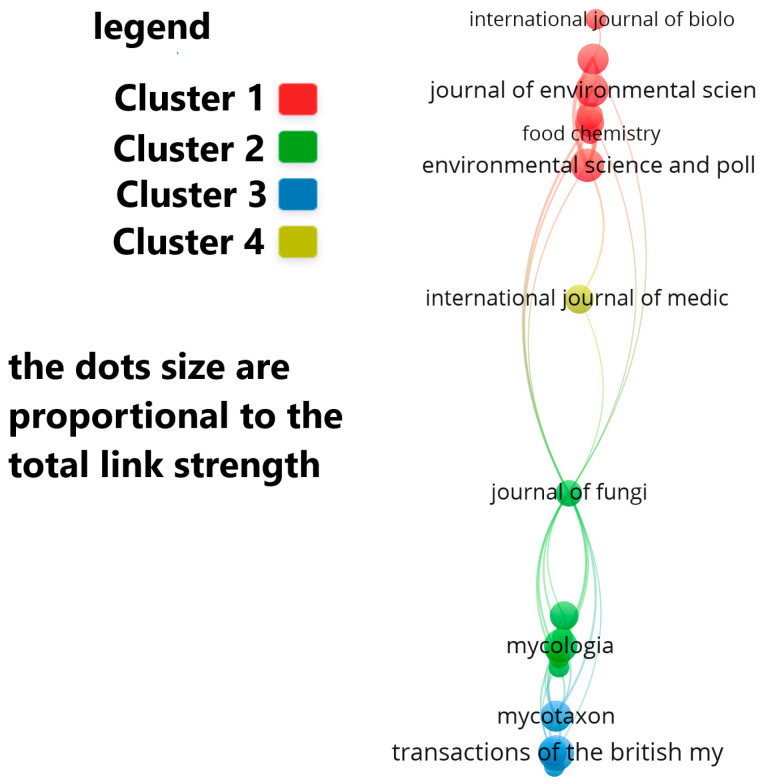
The leading journals that have published articles on *Leccinum*.

Out of the 132 journals that have published articles on this topic, the journals with the largest number of publications are: Environmental Science and Pollution Research, Journal of Environmental Science and Health-Part B-Pesticides foods, Mycologia, Science of the Total Environment and Mycotaxon (Figure 8).

Among the authors who have contributed to these publications, Jerzy Falandysz stands out with 35 articles, followed by Li Tao and Honggao Liu (11 articles each) and Leszek Bielawski (all focused on the chemical composition and nutritional value of wild-grown edible *Leccinum*), as well as Wang Huyiyuan (10 articles). In terms of institutional affiliation, the most representative institutions for authors publishing on this topic were: Fahrenheit University, University of Gdansk, Yunnan Agricultural University and the Chinese Academy of Sciences. The leading publishers in this research domain included Elsevier (46 articles), Springer Nature (39 articles), Taylor and Francis (26 articles) and Wiley (14 articles).

From the analysis carried out on the keywords that appear in the articles published on this topic, it resulted that the most frequently used keywords were: fungi, heavy metals, mushrooms, edible mushrooms and fruiting bodies.

**Figure 9:**
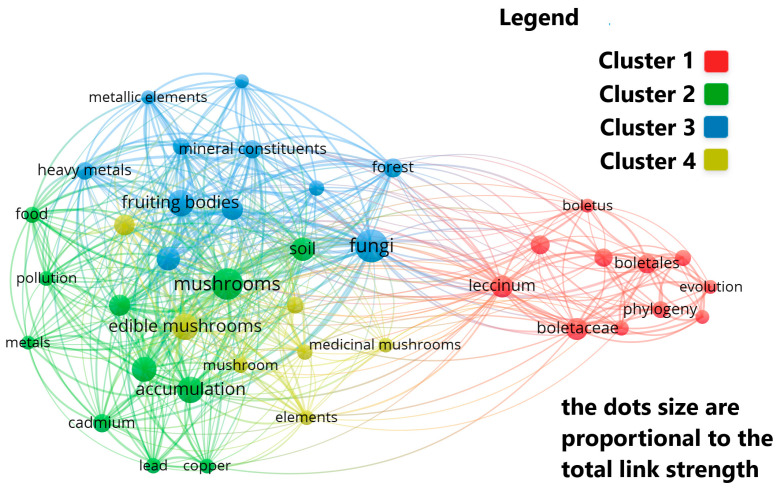
Keywords used by authors in relation to *Leccinum*.

When keywords are grouped into clusters, four main categories emerge, each containing more than six terms: Cluster 1: mainly includes terms related to the genus, species, and taxonomy: Basidiomycota, Boletaceae, Boletales, Boletus, Leccinum, taxonomy. Cluster 2: generally includes terms related to heavy metals: cadmium, copper, heavy metals, lead, mercury. Cluster 3: includes keywords related to minerals and trace elements: mineral constituents, metallic elements, trace elements. Cluster 4: generally includes terms related to medicinal and nutritional values: chemical composition, medicinal mushrooms, nutritional value (Figure 9).

### 3.2. Literature Review

This section synthesizes published data on the genus *Leccinum* to provide an integrated overview of its taxonomic diversity, host associations, physiological traits, nutritional value, and environmental interactions. Rather than reinterpreting results, this section consolidates molecular, ecological, biochemical, and applied studies to highlight recurring patterns and major advances across regions and research approaches. Comparative references to closely related or ecologically co-occurring fungi are retained only where they provide necessary methodological or contextual benchmarks for understanding *Leccinum* biology.

#### 3.2.1. *Leccinum* Species and Their Characteristics

In the Table 1 are presented the distribution, hosts, and characteristics of *Leccinum* species.

Molecular, phylogenetic, and biogeographic studies over the last two decades have fundamentally reshaped the understanding of species diversity and evolutionary relationships within the genus *Leccinum*. Traditional morphology-based sectional classifications have proven inadequate for delimiting natural lineages, and modern multi-locus and genomic approaches reveal a genus characterized by extensive cryptic diversity, host-driven divergence, and complex evolutionary histories.

Early phylogenetic reconstructions based on nrITS and nrLSU sequences demonstrated that historical infrageneric concepts were largely artificial and that *Leccinum* exhibits exceptional ITS length heterogeneity caused by minisatellite repeat regions [39]. Subsequent multilocus and phylogenomic frameworks confirmed *Leccinum* sensu Singer as a monophyletic lineage (with the exception of *L. eximium*) and highlighted the absence of any single diagnostic morphological character defining the genus [57]. These findings established the need for molecular data as the primary basis for species delimitation.

Host specialization has emerged as a central evolutionary driver in *Leccinum*. Most species exhibit strong host specificity, while phylogenetic reconstructions indicate that the broad host range of *L. aurantiacum* represents a derived condition resulting from secondary host expansion [66]. Rapid diversification has been linked to host switching and ecological niche shifts, with hybridization contributing to lineage formation, particularly within section *Scabra* [66]. Phylogeographic analyses further demonstrate that Arctic and boreal lineages are not monophyletic, and only *L. rotundifoliae* shows a strictly Arctic distribution.

Regional surveys and multilocus inventories have substantially expanded the known species pool. In China, early taxonomic uncertainty was resolved through multilocus analyses (nrLSU, rpb2, tef1-α, ITS), which recognized multiple endemic and previously unrecognized species [13,73,88,89]. Comparable progress has been made in Central America [71,90,91], Central Asia [92,93], and Southeast Asia, revealing that *Leccinum* is far more globally diverse than previously assumed.

At a global scale, more than one hundred species are now recognized in North America, with additional centers of diversity in Asia, Europe, Africa, and Oceania [2,37,53]. Nomenclatural clarification of historical names and formal typification of the genus have stabilized taxonomic usage [94,95].

Physiological and population genetic studies further demonstrate high intraspecific diversity and reproductive isolation among cryptic lineages, with evidence for panmixia within populations and strong allozyme variation [96]. Recent whole-genome phylogenies of Boletaceae, including Leccinoideae, place *Leccinum* within a well-supported evolutionary framework [2].

Together, these studies establish *Leccinum* as a phylogenetically complex, globally distributed ectomycorrhizal genus shaped by host specialization, rapid diversification, and repeated geographic radiations.

Collectively, these studies demonstrate that species delimitation in *Leccinum* has shifted from morphology-based concepts toward multi-locus and genomic frameworks, revealing extensive cryptic diversity, frequent host-associated divergence, and occasional hybridization. Regional inventories continue to expand the known species pool, particularly in Asia, the Neotropics, and Oceania, while nomenclatural clarification has stabilized the application of historical names. Together, these findings establish *Leccinum* as a phylogenetically complex and globally distributed ectomycorrhizal lineage, providing the taxonomic foundation for the ecological, nutritional, and applied studies summarized below.

#### 3.2.2. Tree and Plant Species with *Leccinum*

The genus *Leccinum* forms ectomycorrhizal (ECM) associations with a remarkably broad range of woody host plants across boreal, temperate, Mediterranean, and montane tropical ecosystems. Despite this breadth, host specificity remains a defining ecological trait of most species and represents a major driver of diversification within the genus.

Across the Northern Hemisphere, birch (*Betula* spp.) constitutes the dominant host lineage for many *Leccinum* species, including *L. scabrum*, *L. versipelle*, *L. variicolor*, and *L. melaneum* [13,97,98]. Associations with *Populus* species are also widespread, particularly for *L. aurantiacum* and related taxa in Europe and North America [99,100]. These boreal and temperate systems represent the evolutionary core of the genus.

In parallel, *Leccinum* has diversified extensively within the Fagaceae, forming ectomycorrhizae with *Quercus*, *Castanea*, *Castanopsis*, *Cyclobalanopsis*, and *Lithocarpus* across Europe, East Asia, and Southeast Asia [13,101]. These associations extend the genus into subtropical and montane tropical forests and underpin the high species richness observed in East Asia.

More specialized host relationships have also evolved. In North America, *Leccinum* forms ectomycorrhizae with *Hudsonia tomentosa* [43] and ericaceous shrubs such as *Arctostaphylos* and *Comarostaphylis* [17,102,103,104,105,106,107,108,109,110,111,112,113], while conifer associations have been documented with *Picea*, *Pinus*, *Tsuga*, and *Pseudotsuga* [3,105,106].

Collectively, these studies demonstrate that *Leccinum* occupies a broad ecological niche space but remains fundamentally structured by host-driven specialization. This tight coupling between fungal lineages and host phylogeny explains both the high regional endemism and the rapid speciation patterns observed across the genus.

#### 3.2.3. Nutritional Content of *Leccinum*

*Leccinum* species are edible mycorrhizal fungi with both culinary and medicinal potential. Various studies have characterized their chemical composition, bioactive compounds, and flavor-related metabolites, highlighting their value as nutraceutical and functional foods.

Additional compounds include 3,4,5-trihydroxybenzaldehyde and 3,4-dihydroxycinnamic acid in *L. scaber* [107]. Carotenoid analyses showed generally low levels except in *L. duriusculum* [108]. Physiological experiments demonstrated that *L. aurantiacum* alters growth morphology depending on nitrogen availability [106] and can metabolize polycyclic aromatic hydrocarbons [109]. Species-specific germination factors and homing reactions have also been documented in *L. aurantiacum* and *L. versipelle*, with responsiveness linked to homokaryosis [110,111]. Ultrastructural studies of *L. scabrum* revealed complex mycelial strand architecture without thick-walled fibres typical of other fungi [112].

Finally, post-harvest studies showed that drying temperature strongly affects phenolic content, organic acids, ergosterol, and antioxidant activity in *L. scabrum*, with the greatest losses occurring at 70 °C [17].

*Leccinum* species are widely consumed edible ectomycorrhizal mushrooms and represent an important component of traditional diets across Europe, Asia, and North America. Biochemical and metabolomic studies reveal that the genus is characterized by high nutritional value, diverse bioactive metabolites, and species-specific flavor profiles, supporting its classification as a functional and nutraceutical food resource.

Across species, *Leccinum* fruiting bodies are rich in carbohydrates, proteins, dietary fiber, free amino acids, unsaturated fatty acids, vitamins, and essential minerals [12,113]. They also contain a wide range of phenolic compounds, organic acids, carotenoids, and unique fungal pigments such as crocipodin and methyl isoxerocomate [107,108], contributing both to antioxidant capacity and sensory qualities.

Physiological studies further demonstrate that *Leccinum* species exhibit metabolic plasticity in response to nitrogen availability and are capable of degrading complex organic pollutants such as polycyclic aromatic hydrocarbons [106,109]. Post-harvest processing strongly affects nutritional quality, with drying temperature exerting a major influence on phenolic content, ergosterol levels, and antioxidant activity [113].

At the molecular level, metabolomic and transcriptomic profiling of *L. extremiorientale* has identified amino acids and sugars as key determinants of species-specific umami and flavor characteristics [114], while polysaccharides from *L. crocipodium* exhibit prebiotic and short-chain fatty acid–modulating activity in vivo.

Together, these findings position *Leccinum* as a nutritionally rich genus with significant functional food potential, while also highlighting strong interspecific variability in metabolite composition.

#### 3.2.4. Mineral Constituents of *Leccinum*

The mineral composition of *Leccinum* species has been investigated across a wide range of habitats and climatic regions, demonstrating both their nutritional value and their capacity for selective bioaccumulation of essential and potentially toxic elements. Collectively, these studies show that *Leccinum* fruiting bodies are particularly enriched in macronutrients such as K, P, Mg, and Ca, as well as trace elements including Cu, Zn, Mn, Fe, and Rb, while also exhibiting variable accumulation of Cd, Hg, and Pb depending on site conditions.

##### *Leccinum scabrum* 

*Leccinum scabrum* is an edible mushroom commonly found in northern European regions. The macro- and trace-element composition, including Ag, Al, Ba, Ca, Cd, Co, Cu, Fe, K, Mg, Mn, Na, Ni, Pb, P, Rb, Sr, and Zn, was analyzed in both fruiting bodies and underlying topsoil from multiple sampling sites in northern Poland: Darżlubska Wilderness, Trójmiejski Landscape Park, Sobieszewo Island, Wdzydze Landscape Park, and the outskirts of Kętrzyn [115].

Median concentrations in dehydrated caps were highest for K (27,000–44,000 mg kg^−1^), Rb (90–320 mg kg^−1^), and P (6200–9100 mg kg^−1^), followed by Mg (880–1000 mg kg^−1^), Ca (48–210 mg kg^−1^), and Al (15–120 mg kg^−1^). Cu, Fe, Mn, and Zn were detected at 15–27 mg kg^−1^, 38–140 mg kg^−1^, 5.3–27 mg kg^−1^, and 130–270 mg kg^−1^, respectively. Ba and Sr were present at approximately 1 mg kg^−1^ and were nearly equally distributed between caps and stipes. Toxic elements were generally low: Ag (0.48–0.98 mg kg^−1^), Cd (1.0–5.8 mg kg^−1^), Hg (0.36–0.59 mg kg^−1^), and Pb (0.20–0.91 mg kg^−1^), with cap-to-stipe concentration ratios (QC/S) ranging from 1.2 to 4.1. Substantial variations in the pseudo-total (aqua regia extraction) and labile (20% nitric acid extraction) fractions of elements in topsoils were observed between locations. Elements such as K, P, Cd, Cu, Hg, Mn, Na, Rb, and Zn were found to be bioconcentrated in fruiting bodies, though accumulation rates varied with site conditions [115].

A multi-seasonal study using inductively coupled plasma optical emission spectroscopy (ICP-OES) and cold-vapour atomic absorption spectroscopy (CV-AAS) further confirmed these findings, demonstrating wide variations in element concentrations between soil and fruiting bodies over three fruiting seasons. Positive bioconcentration factors (BCFs) were observed for Cd, Cu, Hg, K, Mg, Na, P, Rb, and Zn in caps and stipes, whereas Al, Ba, Ca, Co, Fe, Mn, Ni, Pb, and Sr showed limited accumulation. Caps consistently exhibited significantly different concentrations (*p* < 0.05) of Al, Co, Cu, Hg, Mn, Ni, P, Pb, and Sr across the sampling periods, indicating temporal fluctuations in elemental accumulation [116].

Long-term monitoring of rare earth elements (REEs) in edible mushrooms, including *L. scabrum*, over 45 years across 42 forest sites in Poland revealed a gradual increase in REE content in both mushrooms and soils. Compared to *Boletus edulis* and *Imleria badia*, *L. scabrum* exhibited lower total REE content. Despite these increases, human consumption of *L. scabrum* would not substantially contribute to dietary exposure to REEs, supporting the use of wild-growing mushrooms as bioindicators of environmental REE migration [117].

The most comprehensive mineral profiling has been conducted on *Leccinum scabrum* in northern Poland. Fruiting bodies and underlying soils were analyzed for Ag, Al, Ba, Ca, Cd, Co, Cu, Fe, K, Mg, Mn, Na, Ni, Pb, P, Rb, Sr, and Zn across multiple forest locations [118]. Median concentrations in dehydrated caps were dominated by K (27,000–44,000 mg kg^−1^), P (6200–9100 mg kg^−1^), and Rb (90–320 mg kg^−1^), followed by Mg (880–1000 mg kg^−1^) and Ca (48–210 mg kg^−1^). Trace metals such as Cu, Fe, Mn, and Zn occurred at nutritionally relevant levels, whereas toxic elements (Cd, Hg, Pb, Ag) were generally low. Several elements (K, P, Cd, Cu, Hg, Mn, Na, Rb, Zn) showed clear bioconcentration relative to soils.

A multi-seasonal follow-up study confirmed strong temporal variability and positive bioconcentration factors (BCFs) for Cd, Cu, Hg, K, Mg, Na, P, Rb, and Zn in both caps and stipes, while Al, Ba, Ca, Co, Fe, Mn, Ni, Pb, and Sr showed limited accumulation [116]. Caps consistently accumulated higher concentrations of most elements than stipes.

Long-term monitoring of rare earth elements (REEs) over 45 years across 42 Polish forest sites showed a gradual increase of REEs in *L. scabrum* fruiting bodies, reflecting soil trends. However, dietary exposure from consumption remained negligible, confirming the suitability of *Leccinum* as a bioindicator rather than a dietary risk source [117].

##### *Leccinum versipelle* 

In the Murmansk region (NW Russia), elemental analyses of *Leccinum percandidum* (currently *L. versipelle*) revealed strong accumulation of K and Cu relative to surrounding soils, with K concentrations exceeding soil levels by a factor of 10 and Cu exceeding water-soluble soil Cu by over 70-fold [119]. Nickel showed only limited bioaccumulation. The elemental composition reflected largely background geochemical conditions, highlighting the sensitivity of *Leccinum* species to regional soil chemistry and their role in forest nutrient cycling.

#### 3.2.5. Heavy Metals Content in *Leccinum*

The accumulation of heavy metals in *Leccinum* species has been widely studied in relation to industrial pollution, mining activity, and background geochemistry. Across regions, *Leccinum* consistently demonstrates a capacity to bioaccumulate certain metals—particularly Hg, Cd, Pb, Cu, and Zn—making the genus both a valuable environmental bioindicator and a potential food-safety concern in contaminated areas.

Cadmium and lead

Cadmium (Cd) and lead (Pb) contents were investigated in 699 fruiting bodies of 55 mushroom species collected near a major thermal power plant (Šalek Valley) and an abandoned lead smelter (Upper Meža Valley, Slovenia) [120]. Although the study encompassed a broad fungal assemblage, *Leccinum* species formed part of the edible mushroom group used to assess dietary risk. Elevated Cd and Pb concentrations were associated with polluted sites, and consumption of mushrooms from these areas was identified as a significant human health risk.

Nickel, copper, chromium, and cobalt

In the Kola Peninsula (NW Russia), total concentrations of Ni, Cu, Cr, Sr, As, Pb, Cd, and Co were measured in mushrooms collected between 1987 and 1992, including *Leccinum aurantiacum* and *L. scabrum* [121]. Nickel levels showed a strong correlation with soil concentrations and frequently exceeded food safety limits, rendering mushrooms unsuitable for consumption in affected areas. Other metals generally remained within acceptable ranges, indicating metal-specific accumulation patterns.

Multi-element accumulation in forest plantations

In hybrid aspen plantations under hemiboreal conditions, *Leccinum aurantiacum* fruiting bodies accumulated multiple metals, with mean concentrations reaching 129 mg kg^−1^ Zn, 99 mg kg^−1^ Cu, 30 mg kg^−1^ Mn, 1.5 mg kg^−1^ Ni, 1.7 mg kg^−1^ Cd, 1.1 mg kg^−1^ Cr, and 0.6 mg kg^−1^ Pb [100]. Fertilization with digestate, sewage sludge, and wood ash did not increase heavy-metal uptake, although it altered nitrogen isotopic composition.

Bioaccumulation in relation to soil

In Croatia, five Boletaceae species including *Leccinum* were analyzed for Cd, Cu, Fe, Mn, and Zn [118]. Cadmium accumulation was lower in *Leccinum* (0.73 mg kg^−1^ dw) than in other Boletaceae. Cu and Zn were consistently accumulated across all taxa, whereas Fe and Mn showed species-specific patterns. Only Mn displayed a significant soil–mushroom correlation, underscoring the importance of species-level assessments.

Regional studies in Poland and Eastern Europe

In Poland, *Leccinum aurantiacum* and *L. versipelle* were among seven edible mushrooms examined for Ag, Cd, Cu, Zn, and other elements across rural and industrial regions [122]. Elevated Cd concentrations were observed in *L. versipelle* from the Tarnobrzeska Plain. Similarly, in southwest Poland, *L. scabrum* showed clear accumulation of Ag, Cd, Co, Cu, Hg, Ni, and Zn, with marked species-specific differences [123]. In Ukrainian Polessye, *Leccinum aurantiacum* was among the macromycetes showing the strongest accumulation of Pb and Cd, whereas Cu and Zn were preferentially accumulated by tubular species [124].

Mercury accumulation

Mercury (Hg) is the most intensively studied toxic element in *Leccinum*. In Poland, *L. scabrum* consistently showed higher Hg concentrations in caps than in stems, with BCF values between 9 and 40 [125]. Slovakian surveys of *L. scabrum*, *L. duriusculum*, and *L. albostipitatum* recorded cap Hg concentrations of 0.41–7.52 mg kg^−1^ dw, exceeding EU food limits in some samples [126]. In the Tarnobrzeska Plain, Hg in *L. rufum* reached 3500 ng g^−1^ dw in caps, indicating extreme bioconcentration [127]. Similar accumulation patterns were reported for *L. griseum* [128] and *L. versipelle* [85].

Summary of metal accumulation patterns

Overall, *Leccinum* species exhibit consistent yet highly variable bioaccumulation behavior depending on region, soil chemistry, and species identity. Mercury, cadmium, and lead represent the most critical elements in terms of potential dietary risk, while essential metals such as potassium, copper, and zinc are reliably accumulated and contribute to nutritional value. These characteristics confirm *Leccinum* as a sensitive bioindicator of environmental contamination and a genus requiring site-specific food safety evaluation.

#### 3.2.6. Reactivity Content in *Leccinum*

Studies on radionuclide accumulation demonstrate that *Leccinum* species are highly responsive to regional fallout history, soil chemistry, and habitat type, making them sensitive bioindicators of radiological contamination. Across Europe and North America, accumulation patterns are species-specific and strongly influenced by ecosystem context, particularly in post-Chernobyl landscapes [129,130,131,132,133,134,135,136].

While most regions show radionuclide concentrations well below international safety limits [129,131,135,136], persistent contamination remains evident in parts of Ukraine and Eastern Europe, where *Leccinum scabrum* and related species continue to accumulate elevated levels of ^137^Cs decades after the Chernobyl accident [130,132,133]. Morphological traits such as hymenophore tubule density may serve as phenotypic indicators of radionuclide exposure in bog ecosystems [17].

These data confirm that *Leccinum* species provide valuable long-term records of radionuclide mobility in forest ecosystems.

Overall, radionuclide accumulation in *Leccinum* species reflects strong dependence on habitat type, soil chemistry, and species-specific physiological traits. While contamination levels vary widely across regions, particularly in post-Chernobyl landscapes, most studies indicate that radiological risk from *Leccinum* consumption is highly context-dependent and generally low outside heavily contaminated zones. These compiled data establish *Leccinum* as both a useful bioindicator and a relevant taxon for long-term environmental monitoring.

#### 3.2.7. Utilization of *Leccinum*

Research on *Leccinum* utilization spans food safety, pharmacology, immunology, environmental remediation, and agricultural applications. The studies summarized below document experimentally validated bioactivities and applied functions, providing a descriptive inventory of known uses without extrapolating therapeutic or commercial efficacy beyond reported results.

Traditionally, *Leccinum* species are generally considered edible, but some can cause gastrointestinal issues if not cooked properly. There are reports of *Leccinum* poisonings, so the general recommendation is to cook the mushroom thoroughly; some guides do not recommend certain types, like orange-capped *Leccinum* mushrooms, due to potential toxicity [137,138].

Research on *Leccinum* species demonstrates that members of this genus contain diverse bioactive compounds with pharmacological, immunological, environmental, and plant-physiological relevance.

##### Bioactive Metabolites Affecting Human Health

In a subsequent study, the same species yielded leccinine A, which showed strong endoplasmic reticulum (ER) stress-suppressive activity [139]. ER stress is implicated in several neurodegenerative and metabolic diseases, and leccinine A effectively protected cells from ER stress–induced death. Structural characterization and structure–activity comparisons with seven synthetic analogues confirmed the uniqueness and relevance of this molecule.

*Leccinum scabrum* (birch bolete) contains 5-hydroxytryptophan (5-HTP) and serotonin, which are precursors to the neurotransmitter serotonin in the brain. Since low serotonin levels are associated with depression, consuming mushrooms like *L. scabrum* may be a beneficial dietary component in the prevention or supportive treatment of depression. The potential antidepressant effect of *L. scabrum* is primarily linked to its high content of specific indole derivatives: 5-HTP and L-tryptophan which are converted into serotonin in the central nervous system. *L. scabrum* also exhibits high antioxidant activity due to the presence of phenolic compounds. Oxidative stress and neuroinflammation are thought to play a role in the pathogenesis of depression, so the antioxidant properties of the mushroom may offer protective effects against neuronal damage [76]. Indole compounds and their derivatives (serotonin, melatonin) exhibit antioxidant, anticancer, anti-aging actions, regulate the diurnal cycle in humans and participate in blood coagulation. These compounds and their derivatives are also anti-inflammatory and analgesic therapeutics [140].

*Leccinum scabrum* demonstrated promising antidiabetic potential. Using microwave-assisted extraction and solvents of varying polarity, Ferraro et al. [141] identified EtOAc extracts that potently inhibited α-glucosidase and, to a lesser degree, α-amylase. The extract showed an IC50 for α-glucosidase approximately 60-fold lower than the reference compound 1-deoxynojirimycin. In vivo assays using *Drosophila melanogaster* confirmed the hypoglycemic effects, and metabolic profiling of the active extract was performed by GC-MS and HRMS.

All three polysaccharides enhanced macrophage immunomodulation in vitro, with LCP-2 and LCP-3 producing the strongest responses, suggesting their potential as functional food ingredients or immunomodulatory agents.

Extracts from *Leccinum vulpinum* have also been studied for anticancer properties. Reis et al. [113] examined phenolic extracts rich in hydroxybenzoic acids and found that they inhibited the growth of multiple human cancer cell lines. A more detailed analysis using MCF-7 breast adenocarcinoma cells revealed decreased proliferation, induction of apoptosis, and evidence of DNA damage. MCF-10A non-malignant cells were used as controls to assess selectivity.

##### Immunological Interactions

Jennemann et al. [142] investigated glycoinositolphosphoceramides (GIPCs) from several mushroom species and showed that human sera normally contain IgG2 and IgM heterophile antibodies that recognize basidiolipids from selected taxa, including *Leccinum scabrum*. Recognition depended on the presence of specific Galα1-6Gal or Galβ1-6Man epitopes, and enzymatic removal of these carbohydrate structures abolished immune binding. These findings indicate selective immunological cross-reactivity between human antibodies and certain *Leccinum* lipid components.

*Leccinum vulpinum* have been shown to decrease proliferation and induce apoptosis in breast cancer cells [97,113]. Extracts from *L. scabrum* have demonstrated antitumor activity against cervical, colon, and lung cancer cell lines in lab studies [143]. Extracts from *L. lanipes* can inhibit the cell cycle and induce apoptosis in lung adenocarcinoma (A549) cells [144].

##### Environmental and Agricultural Applications

*Leccinum scabrum* also demonstrated environmental remediation potential. In a study of ectomycorrhizal fungi exposed to the pesticide DDT, Huang et al. [145] showed that *L. scabrum*, along with three other ECMF species, degraded nearly all DDT in liquid culture within 15 days. GC-MS analysis identified metabolites including DDD and DBP, indicating a degradation pathway similar to that of white-rot fungi.

##### Antioxidant and Antimicrobial Properties

Antioxidant Activity

Edible mushrooms, including species of *Leccinum*, are known to contain biologically active compounds such as polyphenols, flavonoids, proteins, saccharides, and vitamins, which contribute to their antioxidant properties.

In another study, the antioxidant activity of methanol extracts of fruiting bodies of *Leccinum duriusculum* was evaluated using high-performance thin-layer chromatography (HPTLC) combined with DPPH assay and videodensitometry. The results indicated that *L. duriusculum* exhibited moderate antioxidant activity compared to *Cyclocybe cylindracea*, and both mushroom extracts had significantly lower activity than ascorbic acid or gallic acid. Specific mushroom components detected in one zone of the *L. duriusculum* extract were mainly responsible for its antioxidant activity [146].

*Leccinum* spp. have a role in anti-aging due to their antioxidant, anti-inflammatory, and immunomodulatory properties, which can combat age-related cellular damage and improve overall health. Key compounds include polysaccharides, phenolic acids, and unsaturated fatty acids, which protect against oxidative stress, promote a healthy gut microbiome, and may offer neuroprotective benefits [66,96].

Antibacterial Activity

The antibacterial potential of *Leccinum scabrum* aqueous extracts was investigated against several bacterial strains. The extracts exhibited inhibitory activity against all tested bacteria. Among the extraction methods, microwave-assisted extraction (MAE) generally showed higher inhibition activity, particularly against *Listeria monocytogenes* ATCC 19114. The Minimum Inhibitory Concentration (MIC) values confirmed the high antibacterial activity of the MAE extract for both *L. monocytogenes* ATCC 19114 and *Escherichia coli* ATCC 25922. In contrast, ultrasound-assisted extraction (UAE) demonstrated strong antibacterial activity against *Salmonella enterica* ATCC 13076 and *L. monocytogenes* ATCC 19114 [12].

## 4. Discussion

### 4.1. Bibliometric Review

The bibliometric analysis shows that original research articles dominate the publication output on *Leccinum*, accounting for more than 90% of all documents. This pattern is consistent with observations from bibliometric analyses in other mycological and ecological fields, where research articles typically represent the majority of publications [147,148,149,150]. However, in contrast to comparable studies in which review papers can approach or exceed 10% of the total output [151,152,153,154], review articles on *Leccinum* account for only about 1%. This disparity highlights a notable lack of integrative syntheses and emphasizes the relevance and timeliness of the present review.

The sharp increase in the number of publications after 2010 follows a trend commonly reported across bibliometric studies in biology and environmental sciences [155,156,157,158]. This growth likely reflects both methodological advances—particularly the widespread adoption of molecular phylogenetics—and an expanding interest in the ecological and applied relevance of fungi. In the case of *Leccinum*, this increased attention is further reinforced by the genus’ importance in ectomycorrhizal ecology, bioaccumulation studies, and food safety research.

Authorship and journal diversity further support the interpretation of a rapidly expanding research field. The large number of contributing authors, their broad geographic distribution, and the wide range of journals involved indicate that *Leccinum* research has moved beyond a regional or taxonomically narrow focus. Similar patterns have been reported in other fungal bibliometric assessments, where expanding collaboration networks coincide with increasing thematic complexity.

Regarding the countries of origin of contributing authors, the strong representation of the United States and China aligns with global publication trends observed in numerous bibliometric studies [57,159,160,161]. However, the prominence of Poland (ranked first) and Japan (ranked fourth) is particularly noteworthy. Both countries have long-standing traditions in mycological systematics and forest ecology, and their leading roles in *Leccinum* research likely reflect sustained national expertise rather than short-term publication trends.

The journals publishing the highest numbers of *Leccinum*-related articles fall into two main thematic categories. Environmental journals—including Environmental Science and Pollution Research, Journal of Environmental Science and Health—Part B, and Science of the Total Environment—primarily feature studies on metal accumulation, pollution monitoring, and food contamination. In contrast, specialist mycological journals such as *Mycologia* and *Mycotaxon* focus on taxonomy, phylogeny, and species descriptions. This dual publication pattern underscores the interdisciplinary nature of *Leccinum* research and its relevance to both fundamental and applied sciences.

Consistent with this observation, keyword analyses reveal a strong and recurring emphasis on heavy metal accumulation, including terms such as “heavy metals,” “mercury,” “accumulation,” and “bioconcentration factor.” The prominence of these terms in *Leccinum*-focused publications highlights the genus’ importance as a model for studying fungal–environment interactions and reinforces its applied relevance in environmental monitoring and food safety contexts.

### 4.2. Taxonomic, Phylogenetic, and Ecological Patterns in Leccinum

Collectively, the reviewed literature demonstrates that *Leccinum* is a morphologically diverse and phylogenetically complex genus whose species boundaries have undergone substantial revision. Early taxonomic frameworks based largely on macromorphological traits proved insufficient, a conclusion now strongly supported by molecular evidence revealing extensive homoplasy and a lack of reliable single diagnostic characters [39,57]. Multilocus phylogenetic analyses using ITS, LSU, rpb2, tef1-α, and Gapdh consistently indicate that many traditional European and North American species concepts require refinement and that cryptic diversity is widespread within the genus [13,67].

Host specificity emerges as a central biological feature shaping *Leccinum* diversification. Most species exhibit strong associations with particular host lineages, suggesting that cycles of host specialization and niche expansion have played a major role in evolutionary divergence. Broad host associations, such as those observed in *L. aurantiacum*, appear to be derived rather than ancestral conditions [39].

Recent studies have also substantially expanded the known geographic range and diversity of *Leccinum*. Previously undocumented species have been described from China, Mexico, Central America, Japan, Australia, and Southeast Asia [13,67]. Many of these taxa exhibit narrow distributions, distinctive colour-change reactions, and unique combinations of microscopic characters, reinforcing the view that global diversity within the genus remains underestimated.

Physiological and biochemical investigations complement taxonomic and phylogenetic findings by revealing functional traits that may underlie ecological success. Studies documenting species-specific germination requirements, PAH degradation pathways, pigment biosynthesis, and nitrogen-responsive growth strategies suggest considerable metabolic versatility [106,107,108,109,110,111]. High allozyme variation and pronounced genetic structuring indicate that many *Leccinum* species maintain large, genetically diverse populations [105], a conclusion supported by recent mitochondrial genome analyses clarifying evolutionary placement within the Boletaceae [106].

Taken together, the continuing discovery of new species, frequent taxonomic reassessments, and the expanding body of molecular, ecological, and biochemical data demonstrate that *Leccinum* remains far from fully resolved. Achieving a stable taxonomic framework will require broader geographic sampling, consistent multilocus approaches, and explicit integration of ecological traits such as host specificity. Similarly, functional studies suggest that *Leccinum* species play significant roles in nutrient cycling, forest symbiosis, and substrate degradation—ecological functions that remain insufficiently explored [17].

### 4.3. Host Specificity and Biogeographic Patterns of Leccinum Ectomycorrhizal Associations

Synthesis of documented ectomycorrhizal associations (Table 1) indicates that *Leccinum* exhibits broad but structured host specificity with clear biogeographic patterns. The strongest signal is associated with *Betula*, which supports the highest diversity of *Leccinum* species across Europe, Asia, and North America, supporting earlier hypotheses that birches represent the ancestral host group for much of the genus [66,98]. The repeated occurrence of *L. scabrum*, *L. versipelle*, and *L. variicolor* on multiple *Betula* species across continents suggests both long-term ecological stability and potential co-migration during postglacial forest expansion.

A second major host axis involves *Populus* species, particularly *P. tremuloides* and *P. tremula*, which support characteristic orange-capped taxa such as *L. aurantiacum* [99,100]. These associations highlight the ability of some *Leccinum* lineages to specialize on fast-growing, disturbance-adapted trees, suggesting an ecological strategy linked to dynamic forest environments [162].

Associations with Fagaceae—including *Quercus*, *Castanea*, and Asian *Castanopsis–Lithocarpus* lineages—indicate diversification alongside major temperate and subtropical oak radiations [13,38,163]. The presence of distinct *Leccinum* assemblages in tropical montane *Quercus* forests of Central and northern South America suggests independent regional radiations within these ecosystems [38].

Although less frequent, records of *Leccinum* associated with ericaceous shrubs and conifers (*Picea*, *Pinus*, *Tsuga*, *Pseudotsuga*) [102,104] demonstrate that some species tolerate a relatively broad range of ectomycorrhizal partners. These atypical associations may reflect ecological plasticity, locally adapted populations, or unresolved cryptic species complexes.

Overall, available evidence indicates that *Leccinum* is primarily associated with Betulaceae, *Populus*, and Fagaceae, while retaining notable ecological breadth. Diversification within the genus appears closely linked to host shifts, geographic isolation, and forest-type specialization. Continued molecular investigation of ectomycorrhizal root tips and integrative taxonomic revision are likely to further expand the known host range and clarify whether currently recognized “generalist” species represent complexes of more specialized lineages.

### 4.4. Insights into the Antibacterial and Antioxidant Potential of Leccinum Mushrooms

Accumulating evidence demonstrates that *Leccinum* species exhibit meaningful antibacterial and antioxidant activities, supporting their growing recognition as potential functional foods and sources of bioactive compounds. Extracts obtained from *L. scabrum* have been shown to inhibit the growth of pathogenic bacteria, reinforcing broader observations that mushrooms produce metabolites with antimicrobial properties. Notably, microwave-assisted extraction (MAE) enhanced antibacterial efficacy compared to conventional methods, particularly against *L. monocytogenes* and *E. coli*, emphasizing the importance of extraction strategy in maximizing bioactivity [12].

The antioxidant capacity of *Leccinum* mushrooms appears to be closely associated with their chemical composition, especially their phenolic compounds, flavonoids, and ascorbic acid content. Variability in radical scavenging activity (RSA) and total phenolics between methanolic and aqueous extracts highlights the strong influence of solvent polarity on the recovery of antioxidant constituents [145]. These findings suggest that differences in reported antioxidant capacity across studies may be partly methodological rather than exclusively species-dependent.

Comparative analyses using HPTLC–DPPH–videodensitometry further contextualize the antioxidant performance of *Leccinum* species. Although *L. duriusculum* demonstrates measurable antioxidant activity, its effectiveness is lower than that of *Cyclocybe cylindracea* and standard reference antioxidants such as ascorbic acid [146]. This comparison underscores that while *Leccinum* mushrooms are promising contributors to dietary antioxidants, their activity is highly species- and compound-specific. Collectively, these studies indicate that *Leccinum* mushrooms possess biologically relevant antibacterial and antioxidant properties, with potential applications in food preservation, nutraceutical development, and health-promoting formulations. Future research should prioritize the isolation of active molecules and optimization of extraction techniques to better harness these properties.

### 4.5. Nutritional and Bioactive Significance of Leccinum Mushrooms

Beyond their bioactivity, *Leccinum* species demonstrate substantial nutritional value, reinforcing their role as multifunctional food resources. Species such as *L. scabrum*, *L. molle*, and *L. vulpinum* are characterized by high carbohydrate and protein contents, favorable profiles of polyunsaturated fatty acids, and appreciable levels of essential vitamins and minerals [12,70]. These compositional features support their inclusion in human diets as sources of both macronutrients and health-promoting compounds.

When considered alongside metabolomic analyses and antioxidant profiles, these results highlight the potential of *Leccinum* species not only as nutritious foods but also as valuable ingredients for functional and culinary applications. The presence of phenolic and organic acids in *L. molle* and *L. vulpinum*, together with documented antioxidant activity, reinforces their broader nutraceutical relevance [70].

### 4.6. Elemental Accumulation, Bioindication, and Nutritional Implications of Leccinum Species

Studies on the mineral composition of *Leccinum* mushrooms reveal pronounced species- and site-specific differences in elemental accumulation, reflecting both ecological function and nutritional significance. In *L. scabrum*, fruiting bodies contain high concentrations of essential macronutrients such as K, P, Mg, and Ca, alongside moderate levels of trace elements including Cu, Fe, Mn, and Zn. At the same time, toxic metals such as Ag, Cd, Hg, and Pb generally occur at low concentrations, indicating minimal risk to consumers under typical conditions [116,117]. Nevertheless, observed variability across locations and seasons highlights the importance of ongoing monitoring.

Element-specific bioconcentration patterns further demonstrate the selective uptake capacity of *L. scabrum*. Positive bioconcentration factors have been reported for K, P, Cd, Cu, Hg, Mg, Na, Rb, and Zn, whereas elements such as Al, Ba, Ca, Co, Fe, Mn, Ni, Pb, and Sr exhibit limited accumulation [116]. These trends align with previous findings and suggest that metal uptake is regulated by both substrate chemistry and environmental conditions [115].

Long-term studies provide additional insight into the bioindicator potential of *Leccinum* species. Temporal and spatial variation in elemental content, including gradual increases in rare earth elements (REEs) over several decades, reflects changing environmental deposition patterns [117]. Importantly, despite these trends, dietary exposure to REEs through consumption of *Leccinum* mushrooms remains negligible.

Research on *L. percandidum* further illustrates the ecological relevance of elemental accumulation. Pronounced enrichment of K and Cu relative to soil concentrations underscores its role in nutrient cycling and highlights its suitability for monitoring environmental health in protected forest ecosystems [119]. The generally low accumulation of Ni and consistency in soil metal content suggest limited anthropogenic impact in studied regions.

Overall, these findings confirm that *Leccinum* species serve as effective indicators of environmental metal availability while simultaneously contributing nutritionally valuable elements to the human diet. Differences among species, anatomical parts, and habitats emphasize the complexity of metal uptake in mushrooms and the necessity of site-specific assessments for accurate evaluations of food safety, ecological monitoring, and human exposure.

### 4.7. Bioaccumulation of Heavy Metals in Leccinum: Implications for Food Safety and Environmental Monitoring

Collectively, the reviewed studies indicate that species within *Leccinum* consistently function as effective bioaccumulators of both essential and toxic metals, although accumulation patterns vary substantially among species and regions. A recurring trend across multiple datasets is the preferential accumulation of metals in caps compared to stems, particularly for Hg, Cd, and Pb [125,126,128]. This organ-specific partitioning has direct implications for human exposure, as caps are typically the primary edible portion.

The elevated accumulation of Cd and Pb in *Leccinum* collected from industrialized or mining-impacted landscapes reinforces the sensitivity of the genus to anthropogenic contamination [120,124]. In several cases, concentrations exceeded recommended safety thresholds, emphasizing that consumption risks are highly site-dependent. Mercury showed particularly strong bioaccumulation, with bioconcentration factors varying widely in response to soil contamination levels and species-specific physiological traits [132]. These findings highlight the need to integrate both environmental context and fungal biology when interpreting metal concentrations in edible mushrooms.

At the same time, *Leccinum* species consistently accumulated nutritionally important elements such as Cu, Zn, and K [118,123]. This dual role—as both nutrient sources and vectors of toxic metals—underscores the complexity of evaluating mushrooms solely as beneficial or hazardous food items. While soil chemistry influenced the uptake of certain elements (e.g., Ni, Cu, Mn), interspecific differences often exerted a stronger effect on accumulation patterns [121,164,165], suggesting genetically mediated uptake mechanisms.

Geographic variation further shaped observed metal loads. Elevated Cd concentrations reported in *L. versipelle* from the Tarnobrzeska Plain, for example, may reflect combined influences of industrial activity and geogenic background levels [106]. Similarly, Hg accumulation in *Leccinum* populations from Poland and Slovakia varied markedly among localities, pointing to heterogeneous contamination sources and the genus’s high accumulation capacity [126,128].

Overall, these patterns confirm *Leccinum* species as useful bioindicators of metal pollution, particularly for Hg, Cd, and Pb. While sporadic consumption from uncontaminated areas likely poses minimal health risk, frequent intake of specimens harvested from polluted environments may result in metal exposure exceeding tolerable limits. Consequently, site-specific monitoring and clear public guidance remain essential for safe foraging practices.

### 4.8. Radionuclide Accumulation Dynamics in Leccinum: Regional Variation and Bioindicator Potential

The reviewed literature demonstrates pronounced regional and ecological variability in radionuclide accumulation by *Leccinum* species, driven by differences in fallout history, soil chemistry, habitat type, and fungal morphology. In regions with low contemporary radionuclide deposition, such as southern Italy, *Leccinum* mushrooms generally exhibit low activities of ^137^Cs and ^40^K, resulting in negligible ingestion doses [129]. In contrast, areas affected by historical nuclear fallout, particularly Ukrainian Polissya, continue to show exceptionally high ^137^Cs levels in several species, including *L. scabrum* [130,132].

Within the genus, species- and organ-specific patterns are evident. Caps frequently contain higher radionuclide concentrations than stems, reflecting differential uptake and translocation processes. This trend is especially pronounced in *L. aurantiacum* and has been consistently reported across multiple studies [133]. Environmental conditions further modulate accumulation, with acidic, organic-rich bog ecosystems promoting enhanced ^137^Cs uptake, as observed in *L. holopus* [17]. The reported correlation between hymenophore tubule density and radiocesium content suggests that morphological traits may serve as additional predictors of radionuclide accumulation [17].

Beyond radiocesium, *Leccinum* species also accumulate naturally occurring radionuclides such as ^40^K and ^210^Po, which contributed most substantially to ingestion doses in northern regions, including Norway [131]. In contrast, uranium- and thorium-series radionuclides generally exhibited low bioconcentration factors and limited relevance for human health [135]. Localized arsenic accumulation near mining areas further illustrates the responsiveness of *Leccinum* to site-specific geochemical anomalies, although much of the arsenic appears to be tightly bound within less bioavailable fractions [134].

Taken together, these findings underscore the value of *Leccinum* as a bioindicator of radionuclide contamination, while also highlighting strong regional contrasts in public health relevance. Whereas mushrooms from Italy and Norway pose minimal radiological risk, those collected in certain Ukrainian and Belarusian regions may still exceed safety thresholds decades after the Chernobyl accident. Continued long-term monitoring and region-specific consumption advisories therefore remain critical.

### 4.9. Biological and Biotechnological Significance of Bioactive Compounds from Leccinum Species

Beyond their ecological role, *Leccinum* species represent a chemically diverse group with emerging relevance in pharmacology, biotechnology, and environmental applications. Studies to date reveal a broad spectrum of bioactive metabolites, including sterols, small secondary metabolites, and structurally complex polysaccharides.

Metabolic regulation is another recurring theme. Extracts of *L. scabrum* demonstrated strong α-glucosidase inhibitory activity, supported by in vivo evidence from Drosophila models [141]. These results suggest that certain *Leccinum* species may contribute bioactive compounds relevant to dietary strategies for glycemic control.

Additional functional properties include antioxidant, antimicrobial, and anticancer activities. Phenolic extracts from *L. vulpinum* induced apoptosis and DNA damage in MCF-7 breast cancer cells [70], while *L. carpini* extracts exhibited antioxidant and antimicrobial effects correlated with phenolic content [148]. Although many of these studies remain limited to in vitro systems, they collectively point to multiple, complementary bioactive compound classes within the genus.

Interactions with the human immune system further extend the biological relevance of *Leccinum*. The selective recognition of glycosylinositol phosphorylceramides from *L. scabrum* by heterophile antibodies in human sera suggests that dietary or environmental exposure to mushroom glycans may have immunological consequences [142].

Finally, environmental and agricultural applications broaden the functional scope of the genus. The demonstrated ability of *L. scabrum* to degrade DDT and generate identifiable metabolites indicates potential for bioremediation [145].

Overall, the literature portrays *Leccinum* as a multifunctional genus with relevance spanning medicine, biotechnology, environmental remediation, and agriculture. While many findings remain preliminary, the diversity of compounds and biological targets provides a strong foundation for future mechanistic and translational research.

### 4.10. Research Gaps and Future Directions

Even though the *Leccinum* genus has shown an increased attention from researchers, there are still significant research gaps, especially related to its evolution, ecology and chemistry.

1. Taxonomy and evolutionary complexity within *Leccinum*

The resolution of leccinoid lineages remains a significant challenge. For example, the boundaries separating *Leccinum* sensu stricto from morphologically similar genera, including *Leccinellum, Hemileccinum, and Garcileccinum*, remain poorly defined. This gap requires genome-scale datasets designed specifically for this taxonomic characters.

Another unexplored aspect is caused by the rapid oxidative colour reactions of *Leccinum*. These vary among lineages and environments, despite their potential utility as taxonomic markers.

In addition, there is a cryptic diversity linked to host specificity. For example, some birch or poplar species can mask host-driven speciation events unique to *Leccinum.* Resolving this hidden diversity will require integrated approaches that synthesize host identity, population genomics, and fine-scale morphology.

2. Host association patterns unique to *Leccinum*

In contrast to most ECM genera, *Leccinum* species commonly exhibit remarkably narrow ecological niches, forming partnerships exclusively with Betulaceae or Salicaceae members. The molecular basis and evolutionary consequences of this fidelity remain unexplained.

Another research gap in this category refers to host switching and postglacial history. For example, Northern Hemisphere *Leccinum* distributions showcase a continuous postglacial expansion alongside specific tree lineages. However, in order to test this hypothesis, a phylogeographic approach must be studied specifically for this genus.

Furthermore, there are some subtropical associations that haven’t been yet explored. For example. the association of *Leccinum* with evergreen Fagaceae and tropical hosts raises important questions about their origin (recent host shifts, overlooked endemic lineages, or ecological convergence.

3. Genus-specific biochemistry and bioactivity

The common bluing and darkening reactions of *Lecciunum* species, when compared with related boletes, signify that this distinct enzymatic system still needs to be studied chemically and genetically. In addition, studies indicate an unusually high variation in trace elements, amino acids, and profiles among *Leccinum* species, yet systematic comparative investigations remain lacking. And let’s not forget the untapped pharmacological relevance of this species. Although bioactivity studies focus on some species, they still have to explore and focus on the unique sterols and phenolic profiles that differ from other Boletaceae.

4. Selective bioaccumulation as a diagnostic trait

*Leccinum* species consistently demonstrate high uptakes of Hg, Cd, and K, often exceeding sympatric ECM fungi. This indicates specific transportation or sequestration mechanisms that need to be further analysed. Furthermore, variation in metal accumulation remains poorly quantified although it can be a unique bioindicator value for this species. Not lastly, despite its importance as a harvested food resource, there are not many studies covering the accumulation of elements during cooking or drying.

5. Applied potential focused on *Leccinum*

*Leccinum* species can be used in bioindication processes and forest monitoring due to their host specificity and predictable accumulation profiles. This usage can be helpful in evaluating forest health and soil contamination, and requires further studies. Furthermore, the species can also be used in restoration processes, as the species is highly dependent on its host. As such, further studies should focus on reforestation programs, especially those targeting birch or poplar systems. And, not lastly, several non-edible *Leccinum* species should be further valorised as they are valuable sources of enzymes, pigments, or metal-binding compounds.

Targeted Future Directions

Future research on *Leccinum* should prioritize genome-scale phylogenesis (in order to solve leccinoid boundaries and species complexes), host connection (especially host fidelity, host-driven speciation, and bioaccumulation analysis linked to host identity), unique aspects to this genus (like its oxidative pigmentation and enzyme systems), as well as its evaluation as a tool useful in bioindication, restoration ecology, and applied mycology.

## 5. Conclusions

This review demonstrates that *Leccinum* is a globally important genus with significant ecological, nutritional, and applied relevance. Bibliometric analysis confirms a steady increase in research output, dominated by studies from Europe, Asia, and North America, but also reveals clear geographic and taxonomic gaps.

Ecologically, *Leccinum* species are key ectomycorrhizal fungi associated with a wide range of woody hosts across diverse biomes. Recent molecular studies have substantially revised species boundaries, exposing cryptic diversity and underscoring the need for continued integrative taxonomic work. Functional studies further indicate marked physiological and biochemical diversity within the genus.

From a human-use perspective, *Leccinum* species represent valuable edible mushrooms with notable nutritional quality and bioactive potential, while simultaneously exhibiting a strong capacity to bioaccumulate heavy metals and radionuclides. This duality highlights their relevance both as functional foods and as bioindicators of environmental contamination.

Overall, the evidence synthesized here identifies *Leccinum* as a promising yet underexplored genus. Future research should prioritize integrative, standardized, and globally coordinated approaches to resolve taxonomy, clarify functional roles, and responsibly develop applications in environmental monitoring, biotechnology, and human health.

## Figures and Tables

**Table 1:** Distribution, hosts, and characteristics of *Leccinum* spp.

Cur. No.	Species	Country	Host	Med	Risk	Citation
1	*Leccinum aberrans* Sm. & Thiers	USA	*Betula*			Tremble et al., 2024 [8]; GBIF, 2023 [37]
2	*Leccinum aeneum* Halling	USA	*Arctostaphylos*			Halling, 1977 [38]; GBIF, 2023 [37]
3	*Leccinum alaskanum* Wells & Kempton	USA	*Betula*			GBIF, 2023 [37]
4	*Leccinum alboroseolum* (Blum) Lannoy & Estadès	AT, BE, DE, FR, IT, SP	*Betula,* broadleaved sp., conifers			GBIF, 2023 [37]
*5*	*Leccinum albostipitatum* den Bakker & Noordel.	BE, DE, DK, FI, FR, IT, MD, NL, NO, RO, RU, SK ^e^, UK	*Populus*		Hg	den Bakker and Noordeloos, 2007 [39]; Šnirc et al. 2023 [40]; Manic, 2015 [41]; GBIF, 2023 [37]
*6*	*Leccinum album* Meng, Li & Yang	CN	*Castanopsis*, *Cyclobalanopsis*, *Lithocarpus*, *Pinus*			Meng et al., 2021 [13]; GBIF, 2023 [37]
7	*Leccinum ambiguum* Sm. & Thiers	USA	*Betula*			Tremble et al., 2024 [8]; GBIF, 2023 [37]
8	*Leccinum anastasiae* Vlasenko	RU	*Salix*			Vlasenko, 2023 [42]
9	*Leccinum angustisporum* Sm., Thiers & Watling	USA				Tremble et al., 2024 [8]; GBIF, 2023 [37]
10	*Leccinum arbuticola* Thiers	MX, USA	*Arbutus*, *Arctostaphylos*			GBIF, 2023 [37]
11	*Leccinum arctoi* Vassilkov	RU				GBIF, 2023 [37]
12	*Leccinum arctostaphyli* Wells & Kempton	USA	*Arctostaphylos*			GBIF, 2023 [37]
13	*Leccinum arenicola* Redhead & Watling	CA, USA	*Hudsonia*			Redhead and Watling, 1979 [43]; Tremble et al., 2024 [8]; GBIF, 2023 [37]
14	*Leccinum areolatum* Smith and Thiers	IN, USA	*Betula*, *Rhododendron*			Semwal et al. 2022 [44]; Tremble et al., 2024 [8]; GBIF, 2023 [37]
15	*Leccinum armeniacum* Thiers	USA	*Arbutus*			GBIF, 2023 [37]
16	*Leccinum aurantiacum* (Bull.) Gray = *Leccinum decipiens* (Singer) Pilát & Dermek; = *Leccinum populinum* Korhonen; = *Leccinum quercinum* (Pilát) Green & Watling; = *Leccinum rufum* (Schaeff.) Kreisel; = *Leccinum salicicola* Watling	AU, BG ^e^, BY ^e^, CA ^e^, CN ^e^, DE, FI, FR, HR, IN, IT, MD, ME, MX ^e^, NL, NO, PL, RO, RU ^e^, SP, UK, USA	*Populus*, *Betula*, *Quercus Fagus*, *Castanea*, *Salix*, *Tilia*	Aox, Atum, Aag, Blcoag, Ainfl, Anlg; Adepr Nutr	Po, Pb, Cd, Se, Mn, U, Th	Hoffeins et al., 2017 [45]; Semwal et al. 2022 [44]; Witkowska et al. 2011 [46]; Tkalčec and Mešić, 2003 [47]; Meng et al., 2021 [13]; Șesan et al., 2010 [48]; Tremble et al., 2024 [8]; GBIF, 2023 [37]; Kuo and Ortiz-Santana, 2020 [3]; Wu et al. 2019 [4]; Binder and Hibbett, 2000 [49]; Vázquez et al., 2016 [50]
17	*Leccinum barrowsii* Sm., Thiers & Watling	CA, MX, USA ^e^	*Pinus*, *Abies*, *Quercus*			Saldivar et al. 2021 [51]; Tremble et al., 2024 [8]
18	*Leccinum boreale* Sm., Thiers & Watling	CA, USA	*Populus*			Schalkwijk-Barendsen, 1991 [52]; Tremble et al., 2024 [8]; GBIF, 2023 [37]
19	*Leccinum borneense* (Corner) Horak	ID, MY	*Betula*			Bánki et al., 2025 [53]
20	*Leccinum broughii* Sm. & Thiers	USA				Tremble et al., 2024 [8]; GBIF, 2023 [37]
21	*Leccinum brunneo-olivaceum* Snell, Dick & Hesler	USA				Tremble et al., 2024 [8]; GBIF, 2023 [37]
22	*Leccinum brunneum* Thiers	USA	*Populus*			Thiers, 1971; Bessette et al., 2000 [54]; GBIF, 2023 [37]
23	*Leccinum californicum* Thiers	USA	*Pinus*			Thiers, 1971; GBIF, 2023 [37]
24	*Leccinum callitrichum* Redeuilh	AT, FI, FR, NO, SE, UK	*Betula*			GBIF, 2023 [37]
25	*Leccinum canumtomentosum* H. Engel	DE				GBIF, 2023 [37]
26	*Leccinum cerinum* M. Korhonen	FI, NO, SE, UK	*Betula*			Korhonen, 1995 [55]; GBIF, 2023 [37]; Binder and Besl, 2000 [49]
27	*Leccinum chalybaeum* Singer	USA	*Quercus*			Bessette et al., 2000 [54]; GBIF, 2023 [37]
28	*Leccinum chioneum* (Fr.) Redeuilh	FR, SP	*Populus*			GBIF, 2023 [37]
29	*Leccinum cinnamomeum* Sm., Thiers & Watling	CA, USA	*Betula*			Tremble et al., 2024 [8]; GBIF, 2023 [37]
30	*Leccinum clavatum* Sm., Thiers & Watling	USA	*Populus*			Tremble et al., 2024 [8]; GBIF, 2023 [37]
31	*Leccinum coloripes* (Blum) Lannoy & Estadès	USA	*Populus*			GBIF, 2023 [37]
32	*Leccinum colubrinum* Sm., Thiers & Watling	USA	*Populus*			Riviere, 2010 [56]; Tremble et al., 2024 [8]
33	*Leccinum constans* Thiers	USA	*Populus*			GBIF, 2023 [37]
34	*Leccinum crocistipidosum* Engel & Dermek	FI, NO, SO, UK				GBIF, 2023 [37]
35	*Leccinum cyaneobasileucum* Lannoy & Estadès = *Leccinum brunneogriseolum* Lannoy & Estadès	AT, CH, DE, DK, FI, FR, MD, NL, NO, PL, SB, SE, SP, UK, USA	*Betula*			den Bakker et al., 2005 [57]; Manic, 2015 [41]
36	*Leccinum disarticulatum* Sm. & Thiers	USA				Tremble et al., 2024 [8]; GBIF, 2023 [37]
37	*Leccinum discolor* Sm., Thiers & Watling	CA, USA ^e^	*Populus*			Bessette et al. 2000 [54]; Tremble et al., 2024 [8]; GBIF, 2023 [37]
38	*Leccinum duriusculum* Schulzer ex Kalchbr. Singer	AT, CH, CZ, DK, FR, HR, HU, MD, ME, NL, PL, RO, RU, SE, SK, SP, UA, UK, UZ	*Populus*	Aox, Hgl, Obes, Nutr	Hg, Po, Pb	Hoffeins et al., 2017 [45]; Lizoň, 2001 [58]; Pál-Fám and Benedek, 2010 [59]; Manic, 2015 [41]; Meng et al. 2021 [13]; Binder and Besl, 2000 [49]; GBIF, 2023 [37]; Tundis et al. 2021 [60]; Jarzyńska and Falandysz, 2012 [61]
39	*Leccinum engelianum* Klofac	DE	*Fagus*			GBIF, 2023 [37]
40	*Leccinum excedens* (Heinem. & Gooss.-Font.) Heinem.	CD, MW, ZM				GBIF, 2023 [37]
41	*Leccinum fallax* Sm., Thiers & Watling	CA, USA				Tremble et al., 2024 [8]
42	*Leccinum fibrillosum* Sm., Thiers & Watling	CA, USA				Tremble et al., 2024 [8]
43	*Leccinum flavostipitatum* Dick & Snell	CA, USA	*Picea*, *Pinus*, broadleaved sp.			den Bakker et al., 2005 [57]; Tremble et al., 2024 [8]
44	*Leccinum floccopus* (Gilbert) Redeuilh	AT, BE, FR, SP, UK				Gargominy, 2025 [62]
45	*Leccinum foetidum* Heinem.	BI, CD, CP, GA, MG, TZ, ZW				GBIF, 2023 [37]
46	*Leccinum fuscescens* Sm., Thiers & Watling	USA				Tremble et al., 2024 [8]
47	*Leccinum fuscoalbum* (Sowerby) Lannoy & Estadès	CH, FR, UK				GBIF, 2023 [37]
48	*Leccinum glutinopallens* Sm., Thiers & Watling	CA, USA				Bessette et al., 2024 [63]; Tremble et al., 2024 [8]
49	*Leccinum griseonigrum* Sm., Thiers & Watling	CA, USA				Bessette et al., 2024 [63]; Tremble et al., 2024 [8]
50	*Leccinum hispanicum* Moreno	SP	*Cistus*			GBIF, 2023 [37]
51	*Leccinum holopus* (Rostk.) Watling = *Leccinum aerugineum* (Fr.) Lannoy & Estadès *= Leccinum nucatum* Lannoy & Estadès; *= Leccinum olivaceosum* Lannoy & Estadès	AT, BY, CA ^e^, CH, CZ, DE, FI, FR, IR, JP, KR, LV, MD, NL, NO, PL, RO, RU, SE, SK, SP, UA ^e^, UK, USA ^e^	*Betula*		Hg, Ce, Pb, Cd, Cu, Zn	Rudawska et al., 2019 [64]; Korhonen, 1995 [55]; den Bakker et al., 2005 [57]; Ivanov, 2013 [65]; Pál-Fám and Benedek, 2010 [59]; Manic, 2015 [41]; Tremble et al., 2024 [8]; Gasecka, 2022 [17]
52	*Leccinum huronense* Sm. & Thiers	USA				Bessette et al. 2000 [54]; Tremble et al., 2024 [8]
53	*Leccinum idahoense* Sm., Thiers & Watling	USA				Tremble et al., 2024 [8]
54	*Leccinum imitatum* Sm., Thiers & Watling	USA				Tremble et al., 2024 [8]
55	*Leccinum incarnatum* Sm., Thiers & Watling	USA				Tremble et al., 2024 [8]
56	*Leccinum indoaurantiacum* Chakr., Das, Baghela, Singh & Dentinger	IN	*Betula*			GBIF, 2023 [37]
57	*Leccinum insigne* Sm., Thiers & Watling	CA ^e^, USA ^e^	*Populus*, *Betula*			den Bakker et al., 2004 [66]; Tremble et al., 2024 [8]
58	*Leccinum insolens* Sm., Thiers & Watling	CA, USA				Bessette et al., 2024 [63]; Tremble et al., 2024 [8]
59	*Leccinum intusrubens* (Corner) Høil.	JP, MY, TH, ZW				GBIF, 2023 [37]
60	*Leccinum juarenzense* Ayala-Vásquez & Pinzón	MX	*Quercus*			Ayala-Vásquez et al., 2023 [67]
61	*Leccinum katmaiense* Wells & Kempton	USA				GBIF, 2023 [37]
62	*Leccinum laetum* Sm., Thiers & Watling	CA, USA				Tremble et al., 2024 [8]; GBIF, 2023 [37]
63	*Leccinum largentii* Thiers	USA	*Arctostaphylos*			Siegel and Schwarz, 2016 [68]
64	*Leccinum leucophaeum* (Pers.) Bon	CA, CH, DE, FR, SP, UK, USA	*Betula*			GBIF, 2023 [37]
65	*Leccinum leucopodium* (Pers.) Dörfelt & G. Berg	BG, BY, CH, DE, DK, EE, FI, FR, GR, IT, LT, NL, NO, PL, RO, RU, SE, SP, UA, UK	*Populus*			den Bakker et al., 2004 [66]; GBIF, 2023 [37]
*66*	*Leccinum longicurvipes* (Snell & Sm.) Kuo & Ortiz	USA	*Quercus*			Kuo and Ortiz-Santana, 2020 [3]; INSDC Sequences 2025 [29]
67	*Leccinum manzanitae* Thiers	CA, NO, USA ^e^,	*Arctostaphylos*			Kuo and Ortiz-Santana, 2020 [3]
68	*Leccinum melaneum* (Smotl.) Pilát & Dermek	AT, CH, CN, DE, DK, LT, NL, NO, PL, SE, SP, RU, UK	*Betula*			Meng et al., 2021 [13]
69	*Leccinum molle* (Bon) Bon	CH, FR, MD, PT ^e^, RO, UK	*Picea*	Aox		Raper, 2023 [69]; Pál-Fám and Benedek, 2010 [59]; GBIF, 2023 [37]; Reis et al., 2016 [70]
70	*Leccinum montanum* Thiers	USA				GBIF, 2023 [37]
71	*Leccinum monticola* Halling & Muell.	CR	*Comarostaphylis*			Halling and Mueller, 2003 [71]; Kuo & Ortiz-Santana, 2020 [3]
*72*	*Leccinum murinaceostipitatum* Sm., Thiers & Watling	USA				GBIF, 2023 [37]
73	*Leccinum murinaceum* (J. Blum) Bon	CH, FR, UK				GBIF, 2023 [37]
74	*Leccinum neotropicale* Halling	CR, GT	*Quercus*			Halling and Mueller, 2003 [71]; GBIF, 2023 [37]
75	*Leccinum nigellum* Redeuilh	FR	*Populus*			Mitchell et al., 2013 [72]; INSDC Sequences 2025 [29]
76	*Leccinum oaxacanum* Ayala-Vásquez, Martínez-Reyes & González-Martínez	MX	*Arbutus*			Ayala-Vásquez et al., 2023 [67]; INSDC Sequences 2025 [29]
77	*Leccinum obscurum* Sm., Thiers & Watling	CA, USA				Tremble et al., 2024 [8]
78	*Leccinum ochraceum* Sm., Thiers & Watling	USA				Tremble et al., 2024 [8]
79	*Leccinum olivaceoglutinosum* Sm., Thiers & Watling	USA				Tremble et al., 2024 [8]
80	*Leccinum olivaceopallidum* Sm., Thiers & Watling	USA				Tremble et al., 2024 [8]
81	*Leccinum onychinum* Watling	CH, NO, UK				GBIF, 2023 [37]
82	*Leccinum pachyderme* (Zeller & Dodge) Kuo & Ortiz	AU, NZ				Kuo and Ortiz-Santana, 2020 [3]; INSDC Sequences 2025 [29]; GBIF, 2023 [37]
*83*	*Leccinum pallidistipes* Sm. & Thiers	USA				Tremble et al., 2024 [8]
84	*Leccinum pallidocastaneum* Wang, Meng, Yang & Li	CN	*Fagaceae*, *Pinaceae*			Wang et al., 2023 [73]
85	*Leccinum parascabrum* Meng & Li & Yang	CN, SB,	*Lithocarpus*, *Castanopsis*, *Pinus*		Fe	Meng et al., 2021 [13]; Dimitrijevic et al. 2014 [74]
86	*Leccinum parvisquamulosum* Dick & Snell	USA				GBIF, 2023 [37]
87	*Leccinum parvulum* Sm., Thiers & Watling	USA				GBIF, 2023 [37]
88	*Leccinum pellstonianum* Sm. & Thiers	USA				Tremble et al., 2024 [8]
89	*Leccinum peronatum* (Corner) Horak	AU				GBIF, 2023 [37]
90	*Leccinum piceinum* Pilát & Dermek	AT, CA ^e^, CH, CZ, DE, FI, FR, HR, IT, NO, RO, SL, SE, SK ^e^, USA	*Picea*		HG	den Bakker et al., 2004 [66]; Šnirc et al. 2023 [40]; GBIF, 2023 [37]
91	*Leccinum phaeocarpum* Wang, Meng, Yang & Li	CN	*Betulaceae*, *Pinaceae*			Wang et al., 2023 [73]
92	*Leccinum ponderosum* Sm., Thiers & Watling	USA	*Pinus*			Tremble et al., 2024 [8]; Koskinen et al., 2019 [75]
93	*Leccinum potteri* Sm., Thiers & Watling	CA, USA				Bessette et al., 2024 [63]; Tremble et al., 2024 [8]
94	*Leccinum proliferum* Sm., Thiers & Watling	USA				Tremble et al., 2024 [8]
95	*Leccinum proximum* Sm. & Thiers	USA				Tremble et al., 2024 [8]
96	*Leccinum pseudoborneense* Meng & Li & Yang	CN	*Castanopsis*, *Lithocarpus*, *Quercus*			Meng et al., 2021 [13]; INSDC Sequences 2025 [29];
97	*Leccinum pseudoinsigne* Sm. & Thiers	CA, USA				Tremble et al., 2024 [8]
98	*Leccinum pulchrum* Lannoy & Estadès	AT, FI, FR, SE, SP, UK				GBIF, 2023 [37]
99	*Leccinum rimulosum* Sm. & Thiers	CA, USA				Tremble et al., 2024 [8]
100	*Leccinum roseoscabrum* Singer & Williams	USA				GBIF, 2023 [37]
101	*Leccinum rubroscabrum* Heinem.	BJ, CP, USA, ZM, ZW				GBIF, 2023 [37]
102	*Leccinum scabrum* (Bull.) Gray = *Leccinum avellaneum* (Blum) = *Leccinum niveum* (Opat.) Rauschert = *Leccinum oxydabile* (Singer) Singer = *Leccinum rigidipes* Orton = *Leccinum roseofractum* Watling = *Leccinum rotundifoliae* (Singer) Sm., Thiers & Watling	AT, BG ^e^, BY ^e^, CA, CH, CZ, CN ^e^, DE, DK, FI, FR, KG ^e^, IN, MD, NL, NO, NZ, PL ^e^, SL ^e^, RO, RU ^e^, SE, SP, UK, USA ^e^	*Betula Fagus*, *Rhododendron*	Abc, Hgl, Aul, Cytox, Aox, Adepr; Nutr	Hg, As, Si	Meng et al., 2021 [13]; Semwal et al. 2022 [44]; Mitchell et al., 2013 [72]; Muszyńska et al., 2013 [76]; Golubkina & Mironov, 2018 [77]; Mirabile et al., 2024 [78]; Chinan, 2011 [79]; Wu et al. 2019 [80]; Orihara et al. 2012 [81]
103	*Leccinum schistophilum* Bon *= Leccinum palustre* Korhonen	AT, BE, CH, CN, DE, DK, EE, FI, FR, JP, NL, NO, RU, SE, UK	*Betula*			den Bakker et al., 2004 [66]; Meng et al., 2021 [13]; Mitchell et al., 2013 [72]; Binder & Besl, 2000 [49]; den Bakker et al., 2005 [57]; Raspé et al. 2016 [10]; Ivanov, 2013 [65]; INSDC Sequences [29]
104	*Leccinum singeri* Sm. & Thiers	USA				Tremble et al., 2024 [8]
105	*Leccinum snellii* A.H. Sm., Thiers & Watling	CA ^e^, USA	*Betula*			den Bakker et al., 2004 [66]; Kuo & Ortiz-Santana, 2020 [3]; Tremble et al., 2024 [8]
106	*Leccinum solheimii* Sm., Thiers & Watling	USA				Tremble et al., 2024 [8]
107	*Leccinum squarrosipes* (Corner) Horak	MY				GBIF, 2023 [37]
108	*Leccinum subalpinum* Thiers	USA				GBIF, 2023 [37]
109	*Leccinum subatratum* Sm., Thiers & Watling	USA				Tremble et al., 2024 [8]
110	*Leccinum subfulvum* Sm., Thiers & Watling	CA, USA				Tremble et al., 2024 [8]
111	*Leccinum subgranulosum* Sm. & Thiers	CA, USA				Tremble et al., 2024 [8]
112	*Leccinum subleucophaeum* Dick & Snell *L. s.* var. *minimum*	CA, CN, TK, USA				Meng et al., 2021 [13]; GBIF, 2023 [37]
113	*Leccinum sublutescens* Sm., Thiers & Watling	USA				Tremble et al., 2024 [8]
114	*Leccinum subpulchripes* Sm. & Thiers	CA, USA				Tremble et al., 2024; [8]
115	*Leccinum subradicatum* Hongo	JP				GBIF, 2023 [37]; Wu et al., 2019 [80]
116	*Leccinum subrobustum* Sm., Thiers & Watling	USA				Tremble et al., 2024 [8]
117	*Leccinum subrotundifoliae* (Blum) Bon					GBIF, 2023 [37]
118	*Leccinum subspadiceum* Sm., Thiers & Watling	USA				Tremble et al., 2024 [8]
119	*Leccinum subtestaceum* Sm., Thiers & Watling	CA, USA				Tremble et al., 2024 [8]
120	*Leccinum succineobrunneum* Dick & Snell	USA				GBIF, 2023 [37]
121	*Leccinum tablense* Halling & Muell.	CR	*Quercus*			Halling and Mueller, 2003 [71]; Kuo & Ortiz-Santana, 2020 [3]
122	*Leccinum talamancae* Halling, Gómez & Lannoy	CR	*Quercus*			Halling & Mueller, 2003 [71]
123	*Leccinum tenax* Heinem.	CD	*Quercus*			GBIF, 2023 [37]
124	*Leccinum thalassinum* Pilát & Dermek	SK				Lizoň, 2001 [58]
125	*Leccinum tlemcenense* (Maire) Redeuilh	CR	*Quercus*			Halling and Mueller, 2003 [71]
126	*Leccinum truebloodii* Sm., Thiers & Watling	CR, USA	*Quercus*			Halling and Mueller, 2003 [71]; Tremble et al., 2024 [8]
127	*Leccinum uliginosum* Sm. & Thiers	CA, USA	*Populus*			den Bakker et al., 2004 [66]; Tremble et al., 2024 [8]
128	*Leccinum umbonatum* Heinem.	BJ, CD, MW				Bánki et al., 2025 [53]
129	*Leccinum umbrinoides* (Blum) Lannoy & Estadès					Bánki et al., 2025 [53]
130	*Leccinum ustale* (Berk.) Horak	AT, CH, DE, FR, IN, PK, SE, SP, UK				Verma and Pandro, 2018 [82]
131	*Leccinum variabile* Sm., Thiers & Watling	USA				Tremble et al., 2024 [8]
132	*Leccinum variicolor* Watling	AT, BE, BY, CA, CH, CZ, DE, EE, FI, FR, IR, IS, JP, LT, LV, MD, ME, NL, NO, RO, RU, SE, SK, SL, SP, UA, UK, USA	*Betula*			Mitchell et al., 2013 [72]; Binder and Besl 2000 [49]; Den Bakker et al., 2005 [57]; Ivanov, 2013 [65]; Tremble et al., 2024 [8]; Chinan, 2010 [83]; Manic, 2015 [41]
132	*Leccinum variobrunneum* Dick & Snell	USA				Bánki et al., 2025 [53]; GBIF, 2023 [37]
134	*Leccinum versipelle* (Fr. & Hök) Snell *= Leccinum atrostipitatum* Sm., Thiers & Watling *= Leccinum percandidum* Lannoy & Estadès = *Leccinum testaceoscabrum;* *= Leccinum atrostipitatum*; = *Leccinum rufescens* (Secr. ex Konrad) Šutara = *Leccinum roseotinctum* Watling	PL ^e^, NL, BE, FR, UK, IR, DE, NO, SE, FI, EE, LV, LT, AT, CZ, SK, SL ^e^, SB, BG, RO, MD, UA, RU ^e^, BY, TR, USA, CN, CA ^e^, JP, RK, IN AT, BE, BG, BY, CA ^e^, CN, CZ, DE, EE, FI, FR, IN, IR, JP, LT, LV, MD, NL, NO, PL ^e^, RK, RO, RU ^e^, SB, SK, SL ^e^, SE, TR, UA, UK, USA	*Betula* *Arctostaphylos*	Aox, Nutr	Cu, Hg, Pb	Keles et al., 2017 [84]; Mirabile et al., 2024 [78]; Ivanov, 2013 [65]; Krasińska & Falandysz, 2016 [85]; Meng et al. 2021 [13]; Kuo and Ortiz-Santana, 2020 [3]; Mitchell et al., 2013 [72]; Tremble et al., 2024 [8]; Ciortan and Negrean, 2013 [86]; Manic, 2015 [41]; Niedzielski et al. 2024 [87]
135	*Leccinum vinaceopallidum* Sm., Thiers & Watling	USA				GBIF, 2023 [37]
136	*Leccinum violaceotinctum* Ortiz & Baroni	BZ, CU	*Quercus*, *Pinus*			Kuo and Ortiz-Santana, 2020 [3]
137	*Leccinum vulpinum* Watling	HR, JP, ME, NO, PT ^e^, RO, UK	*Pinus*	Aox, Apr, Nutr	Po, Pb, U, Th	den Bakker et al., 2004 [66]; Reis et al., 2016 [70]

**Country**: country name abbreviation; ^e^—edible; Med: medicinal use; Aox: antioxidant, Apr: antiproliferative, Atum: antitumour; Abc: antibacterial; Anlg: analgesic; Adepr: antidepressive; Hgl: Hypoglicemic; Aag: anti-aging; Blcoag: blood coagulation; Aul: antiulcer, Ainfl: anti-inflammatory; Cytox: cytotoxic; Obes: obesity; Nutr: Nutrition; Risk: risk of contamination with chemical elements (Hg-mercury, Po-polonium, etc.).

## Data Availability

No new data were created or analyzed in this study. Data sharing is not applicable to this article.
